# Evaluating the Effectiveness of a Roblox Video Game (Super U Story) in Improving Body Image Among Children and Adolescents in the United States: Randomized Controlled Trial

**DOI:** 10.2196/66625

**Published:** 2025-07-31

**Authors:** Nicole Paraskeva, Sharon Haywood, Jason Anquandah, Paul White, Mahira Budhraja, Phillippa C Diedrichs, Heidi Williamson

**Affiliations:** 1 Centre for Appearance Research University of the West of England Bristol United Kingdom; 2 Department of Engineering, Design and Mathematics University of the West of England Bristol United Kingdom

**Keywords:** randomized controlled trial, microintervention, Roblox, video game, gaming, body image, children, adolescents, preadolescents, body satisfaction

## Abstract

**Background:**

Body dissatisfaction is a global public health issue negatively impacting young people’s mental and physical well-being, underscoring an urgent need to develop early interventions. Emerging evidence suggests that microinterventions are acceptable and effective in delivering mental health interventions. Given the popularity of video games among young people, gaming holds great promise for body image microinterventions. As such, we developed Super U Story, a stand-alone, self-paced, narrative-based adventure video game for the popular gaming platform Roblox grounded in the Tripartite Influence Model of body dissatisfaction and basic tenets of positive body image.

**Objective:**

This trial evaluated the effectiveness of playing a purpose-built Roblox video game once on US children and adolescents’ state and trait body image and related outcomes. Gameplay was capped at 30 minutes.

**Methods:**

Overall, 1059 US-based girls and boys (n=460, 43.4% girls) aged 9 to 13 years (mean age 10.9, SD 1.36 years) from diverse ethnic, socioeconomic, and geographic backgrounds were recruited online via a research agency into a 3-arm, online, parallel randomized controlled trial. Participants were assigned to an intervention group, active control group (a Roblox game called Rainbow Friends 2 Story [Color Story]), or attention control group (web-based word search). Participants completed self-report assessments at baseline (1 week before the intervention and before randomization), immediately before and after intervention testing, and 1 week after the intervention. Outcomes included state measures of body satisfaction (primary outcome), mood, and body functionality and trait measures of body esteem, body appreciation, internalization of appearance ideals, and social media literacy. Data were evaluated using repeated-measure analysis of covariance controlling for baseline. Engagement and acceptability data were collected.

**Results:**

Intervention participants showed improved state body satisfaction (*F*_1,694_=5.20; *P*=.02; η_p_^2^=0.01) relative to the active control but not in comparison to the attention control. State mood, state body functionality, internalization of appearance ideals, and social media literacy showed no effects. Relative to the intervention group, the active control showed improved trait body esteem (*F*_1,663_=5.40; *P*=.02; η_p_^2^=0.01) and body appreciation (*F*_1,663_=6.08; *P*=.01; η_p_^2^=0.01). Exploratory analyses found that age and gender did not moderate the effects. We were unable to examine dose-response effects. Acceptability scores were good. Self-report engagement data suggested that participants experienced a highly variable and often low-dose exposure.

**Conclusions:**

This large-scale, fully powered trial is the first to assess the effectiveness of a Roblox-based body image intervention, demonstrating the potential for disseminating microinterventions to children and adolescents on large and popular commercial platforms. Overall, playing Super U Story did not cause harm; however, evidence is lacking to suggest that it improved body image. Learnings are discussed, including psychoeducation as an intervention technique, “chocolate-covered broccoli” phenomena (ie, losing players who recognize thinly disguised educational messages), and measuring intervention engagement.

**Trial Registration:**

ClinicalTrials.gov NCT05669053; https://clinicaltrials.gov/study/NCT05669053

## Introduction

### Background

Body image issues prevalent during pre- and early adolescence negatively impact the mental and physical health of young people [1-4]. For example, prospective studies have established that body dissatisfaction during later childhood and early adolescence predicts dieting behaviors and reduced physical activity [5], depression [6], poor self-esteem [7], and eating disorders [8]. Although most body image interventions aimed at young people focus on adolescents [9-11], up to half of children aged 6 to 12 years are unhappy with their appearance [2], suggesting that more interventions aimed at younger cohorts to prevent or reduce poor body image and promote positive body image are vital [5,11]. In addition, the prospective evaluation by Lacroix et al [12] of body esteem development in girls and boys aged 11 to 15 years found that, among those with low body esteem, dissatisfaction was entrenched by the age of 11 years and remained stable throughout adolescence, which further supports the need for earlier intervention before poor body image becomes established.

Given the widespread nature of body image issues among young people, traditional approaches tend to be used, namely, interventions based in schools [10], the community, or clinical environments [13]. However, substantial barriers to the dissemination and acceptability of face-to-face interventions exist, including the global lack of human resources required to deliver interventions [14], the costs involved [15], and the stigma attached to mental health issues [15,16]. As a result, other approaches need to be explored to reach young people at scale. In particular, a focus on prevention or low-level concerns (eg, mild to moderate discontent related to one’s appearance that does not impact day-to-day living, such as opting out of activities or engaging in body-changing strategies) requires fewer resources and could help reduce the strain on the mental health care system through intervention in digital or community settings [17,18].

Much evidence has shown that interventions targeting universal, nonclinical samples of young people have been successful in reducing body image concerns [19-22]. One such approach is through the use of microinterventions. Microinterventions offer a lighter touch in comparison to traditional interventions. They are designed to be self-guided and of short duration to elicit immediate improvements in the targeted symptoms [23]. Furthermore, microinterventions may be delivered only once [24,25] or repeatedly [23,26]. Given the brevity and self-guided nature of microinterventions, they are well suited to digital environments [27], such as via videos [23,28], social media [29], and online games [30]. To date, we are aware of only 2 rigorously tested body image microinterventions aimed at children—a 60-second psychoeducational cartoon and a 120-second playable (ie, in-app) interactive game for adolescents aged 13 and 14 years—both of which demonstrated improvements in state body satisfaction [30,31]. Similarly, a body image chatbot for adolescents aged 13 to 18 years containing various microinterventions proved effective at improving both state and trait body image [32].

Given the encouraging results from Matheson et al [30] and the wide scope and popularity of video games among young people [33], the online gaming world holds great promise for housing body image microinterventions. Researchers have been tapping into the use of online games to deliver mental health interventions for several years [34-36]. However, other than the playable intervention by Matheson et al [30], to our knowledge, no other online game designed to improve body image has attempted to enter the gaming landscape. As such, this paper focuses on Super U Story, an innovative stand-alone video game microintervention embedded within the Roblox gaming platform intended to reduce body dissatisfaction and promote positive body image among girls and boys aged 9 to 13 years. The Roblox gaming platform houses a huge array of online games aimed at young people, most of which are free to play, created by both gaming developers and users alike for the purposes of entertainment [37] (ie, it is generally not used for educational or intervention content). Although no academic literature exists regarding the impact of Roblox games on body image and related outcomes, this platform was chosen because of its potential to reach millions of children; as of 2024, Roblox had >216 million active monthly users worldwide, with almost an equal number of female and male players, and 42% were aged <13 years [38]. As is typical of Roblox games, Super U Story combines both mandatory and nonmandatory content to improve body image in its players embedded in a dramatic narrative, a key element in video games promoting health-related behavior change [39].

### Super U Story

Super U Story is grounded in the tripartite influence model of body dissatisfaction [40] and the tenets of positive body image [41]. The Tripartite influence model addresses well-established risk factors for negative body image, namely, internalization of appearance ideals (ie, personal belief in society’s standards of beauty and taking action to try to achieve the ideal appearance) [40] and appearance-based social comparisons (ie, making appearance-based comparisons with others) via the sociocultural influences of the media, peers, and family. Super U Story incorporates 2 of these influences—media and peers—with a focus on media, which encompasses both traditional and social media [42,43] and is considered to be the most pervasive [44]. These risk factors are targeted through psychoeducation that shares strategies to manage appearance-related teasing and bullying, elucidates the harms of engaging in appearance-based comparisons, and promotes social media literacy (ie, skills related to critical thinking and skepticism regarding social media content and the motivations for creating such content) [45,46]. Given social media’s focus on appearance and its prolific content and messaging regarding achieving an “ideal” body [47], targeting these risk factors was especially pertinent. Furthermore, evidence exists suggesting that facilitating social media literacy skills in young people plays a protective role against body dissatisfaction [45,46]. Although body image interventions have historically focused on reducing established risk factors [10], there is a growing trend to address and reinforce body image–related strengths and protective factors [48] in interventions. Specifically, tapping into positive body image—a construct involving various facets independent of negative body image, namely, an appreciation of and respect for one’s body and its functions regardless of whether it conforms with society’s beauty standards—shows promise [49]. Positive body image has been associated with psychological well-being (eg, positive affect, high self-esteem, and optimism) as well as healthy behaviors such as increased engagement with exercise, positive self-care behaviors (eg, protecting the skin from sun damage and engaging in regular oral health habits), and intuitive eating [49]. For this reason, Super U Story also applies tenets of positive body image by focusing on bolstering protective factors known to promote positive body image, including body functionality (ie, valuing one’s body for what it can do instead of for how it looks), body appreciation (ie, respecting and caring for the body), and an appreciation of diverse body shapes and features [50,51]. Targeting protective factors during childhood could reduce the likelihood of body dissatisfaction developing during early adolescence [4,52]. Similar to the approach taken with risk factors, psychoeducation is used in Super U Story to promote the 3 aforementioned protective factors.

### This Study

This 3-arm, online randomized controlled trial (RCT) evaluated the effectiveness of Super U Story in eliciting immediate and short-term improvements in children’s and adolescents’ state- and trait-based body image and related outcomes.

This study aimed to test four hypotheses:

1. Participants randomized into the Super U Story condition will experience greater improvements in state-based body satisfaction (primary outcome), body functionality, and mood immediately following the intervention (eg, postintervention assessment) relative to the 2 control conditions (attention and active control).
2. Participants randomized into the Super U Story condition will experience improved trait-based body esteem, body appreciation, and social media literacy and a reduction in internalization of appearance ideals at the 1-week follow-up relative to the 2 control conditions (attention control and active control).
3. Intervention effects will be moderated by gender and age. Specifically, it is hypothesized that intervention effects will be greatest among girls, as well as among girls and boys aged 12 to 13 years (vs those aged 9-11 years). Previous research suggests that intervention effects are moderated by gender [53], and given that body image issues are considered most salient in adolescence [54], it is expected that older boys and girls aged 12 to 13 years will experience the greatest benefit. These analyses are exploratory.
4. It is expected that greater engagement with the key messaging and activities in the intervention condition will result in greater improvements in state- and trait-based outcomes. This dose-response analysis will be exploratory due to the novelty of this intervention.

## Methods

### Trial Design

This study was a 3-arm, online, parallel RCT conducted in 2023 in the United States to evaluate the effectiveness of Super U Story using an intervention group, an active control group, and an attention control group. Participants were randomized to a group using the least filled quota to ensure an even distribution of age, gender, socioeconomic status, and ethnicity across the 3 groups. The feasibility of the study was first tested in a pilot study.

### Ethical Considerations

This study received ethics approval from the Health and Applied Sciences Research Ethics Committee at the University of the West of England, Bristol (HAS.22.11.040) and was preregistered with ClinicalTrials.gov (NCT05669053). Before recruitment, informed written consent was obtained from parents or guardians. Consent forms outlined who was conducting the study, the aim of the research and what it entailed, and the benefits and possible risks. It also ensured the highest level of confidentiality regarding the information shared by the participants, explaining that a unique participation code was assigned to each participant so that participants could not be identified. The complete anonymity of the participants was guaranteed regarding any reports or publications on this study’s results. The voluntary nature of their children’s participation was underscored, and the participant, parent, or guardian had the right to withdraw from the study at any time without providing a reason. Finally, parents or guardians could request the removal of their children’s data from the study up until the point of data analysis. With regard to compensation, participants were sent incentives totaling US $40 (US $10 after the first survey, US $15 after the second survey, and US $15 after the final survey). They were also entered into a sweepstake (free prize draw) to win 1 of 3 US $1000 prizes in the form Visa gift card claim codes.

### Participants

The participants were recruited and enrolled via a US-based research agency. A sample of computer- and internet-literate girls and boys from diverse ethnic, socioeconomic, and geographic backgrounds (ie, from all 50 states) were recruited. Eligible participants were US-resident girls and boys aged 9 to 13 years who played Roblox games a minimum of 4 hours per week. They were excluded from the study if they had previously played the Super U Story game or if their parent or guardian did not provide written consent.

The research agency recruited participants online using their existing databases of adult participants and sample partners to ensure national representation. Sample partners are companies who own and manage panels with respondents who opt in to take surveys; these companies have been fully vetted as trusted and reliable sample suppliers by the agency’s field management team. In all cases, the sample was opt in, meaning that respondents had opted in to receive surveys from the research agency. The young people recruited into this trial were originally sourced through their parents or guardians. The research agency sent those parents or guardians who had at least one child aged between 9 and 13 years a link to a study information sheet and screening questionnaire. If a household had multiple children who met the inclusion criteria, the agency selected 1 child based on the age and gender most needed to meet age and gender quotas. Parents or guardians supplied demographic information on behalf of their children (ie, socioeconomic status, ethnicity, age, and gender) and completed the screening questionnaire to determine whether they met the inclusion criteria. Following the screening questionnaire, parents or guardians provided written consent. In addition, at the beginning of each of the study’s questionnaires, participants had the opportunity to read the study information (including their right to withdraw without penalty) and provide written assent. Participants were instructed to complete the study questionnaires on the device they usually played games on (ie, mobile phone, laptop or desktop computer, or tablet).

The research agency took several measures to ensure that the participants were quasi-anonymous and could not assume multiple identities. Their sample partners assigned each respondent a unique and anonymous ID number to safeguard against a respondent being screened more than once, and they used MyCleanID (RealDefense LLC) [55] and digital fingerprinting technology (ie, FingerprintJS) [56] to identify respondents’ devices and track survey interactions. Furthermore, each respondent was assigned a unique questionnaire link, which became inactive after it had been completed once to prevent duplicate submissions.

### The Intervention

**Figure 1:**
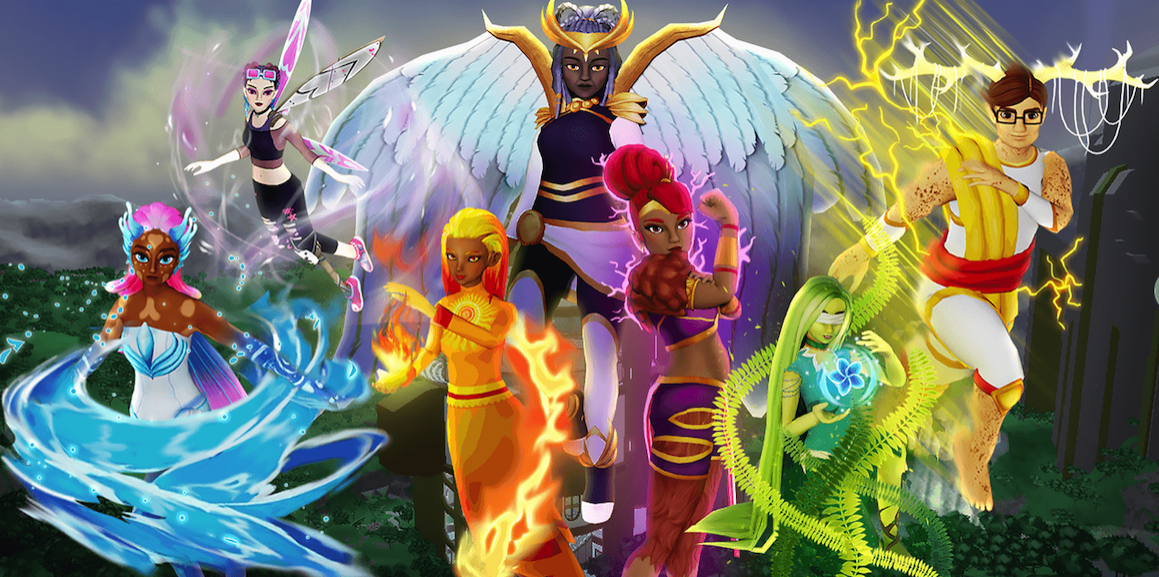
Image from Super U Story featuring the game’s trainer characters.

Super U Story was created through an industry-academic partnership between Toya, a gaming studio that creates video games for the online gaming platform Roblox, and the Dove Self-Esteem Project, the social mission for Unilever’s personal care brand, Dove, in consultation with the authors of this paper (ie, body image researchers). Toya owns the intervention. The intervention (version 1; March 4, 2023) is a narrative-based adventure video game designed for the Roblox platform aimed at children aged 9 to 13 years. Minor changes to character dialogue in 4 short scenes with nonplayer characters were implemented after the trial commenced (version 2; March 17, 2023). Playing Super U Story from start to finish takes approximately 20 minutes. The storyline was initially developed by Toya, which centers on The Academy, a school for kids with developing superpowers that comes under attack by a group of rogue ex-students intent on spreading negativity. As players progress through the game, navigating obstacles, learning about the unfolding narrative, and strengthening their newly acquired superpower to save The Academy, they are exposed to psychoeducational content through pop-up messages housed in a fictional social media platform named Flutter, nonplayer characters’ dialogue and conversations, interactive conversations between the players and nonplayer characters, and dialogue from The Academy’s trainers (Figure 1). Players reaching the end of the game are presented with 3 possible endings, 2 of which involve defeating the threat to the school. All 3 possible conclusions reinforce the psychological messages embedded throughout the game’s journey.

The development of Super U Story was guided by best practice for games that promote health outcomes, which recommends collaboration between game designers and academics to ensure that the game is grounded in evidence and theory [57]. As such, authors NP, HW, SH, and PCD provided Toya with recommendations and rationale related to theoretical content, such as the wording of key messages to target risk factors associated with the Tripartite influence model [40] and the protective factors connected to positive body image [41]. This included the development of the game’s characters to ensure a wide assortment of body types to encourage an appreciation of various kinds of bodies, considering body size, ethnicity, hair type, ability, facial and bodily features, and appearance-affecting conditions such as vitiligo. Drawing on their expertise, the game developers at Toya identified restrictions regarding how and where educational messages could be incorporated to maximize the length of time that players engaged with the game. A primary concern of Toya, and of developers more broadly, is to avoid their games appearing to be “edutainment,” thinly disguised educational software or “chocolate-covered broccoli,” an approach proven to be ineffective with children [58,59]. Toya’s valid concern about losing players who recognize and object to this technique needed to be balanced with embedding evidence-based psychoeducation in an activity that young people choose to engage in for purely entertainment purposes. As a result, most of the intervention’s content was embedded in gameplay options that were nonmandatory for players, a key feature of Roblox games that gives players the freedom to pick and choose the elements with which they wish to interact (Multimedia Appendix 1).

Toya engaged in their typical approach to user testing throughout the game development process, which involved regular testing with their user testing community via private servers on the Discord platform [60]. Authors NP, HW, and SH were invited to view 3 recorded sessions conducted on June 25, 2022; July 2, 2022; and July 23, 2022, for which the authors provided a set of questions for the sessions’ facilitator to ask the players regarding comprehension of the game’s narrative and key psychoeducational messages. Participants consisted of 33 regular Roblox players in the United States ranging in age from 7 to 17 years. The privacy of Discord’s user testing community is protected; as such, further demographic information for these players was not made available. In addition, the authors shared a short survey on the survey platform Qualtrics (Qualtrics International Inc) to assess Roblox players’ comprehension and acceptability of Super U Story with 4 young people in the United States ranging in age from 9 to 13 years (mean age 10.5, SD 1.91 years) recruited through convenience sampling (n=3, 75% boys; n=1, 25% preferred not to say). Following these 3 user testing sessions and receipt of the survey results, the game developers at Toya and authors NP, HW, and SH worked together to edit the game’s narration and dialogue to maximize players’ full comprehension of the storyline and psychoeducational messages. Multimedia Appendix 1 provides an intervention summary.

To isolate the key components of the intervention, Super U Story was compared to 2 control conditions: an alternative Roblox game (active control) and a web-based word search (attention control), described in the following sections.

### Active Control

The active control condition was assigned to play the Roblox game Rainbow Friends 2 Story (Color Story), a similar Roblox game that was not intended to target the outcomes of interest (ie, body image and related outcomes, the active ingredients of the intervention [61,62]). It should be noted that, following the trial, the name of this game was updated to “Colors (Story),” but the narrative and key game elements remained the same. As recommended when choosing an active control [63], other key features of Rainbow Friends 2 Story (Color Story) were evaluated to be a close match with those of Super U Story—both games are based on a similar narrative (ie, saving the world or The Academy), have the same approximate duration of gameplay, present challenges and obstacles to tackle, and have various interactive nonmandatory elements with which to engage.

### Attention Control

The attention control condition was assigned an age-appropriate online word search where participants were required to find words related to animals [64] in as many word searches as they wished to engage with for up to 30 minutes, the maximum amount of time that participants in the intervention and active control conditions were instructed to play. The purpose of the attention control condition was to ensure that participants were provided with an activity of a similar duration as that of the intervention to occupy their time and attention but without any other components featured in the intervention [62,65]. In addition, given the physicality of gameplay in Roblox games (ie, avatars controlled by the player are in almost constant motion, engaging in movement such as running, jumping, and swimming, particularly when navigating obstacles), it is possible that this aspect could impact body functionality. As such, we included an attention control condition featuring a non-Roblox game to account for this potential effect.

### Procedure

The researchers had no contact with the participants during any phase of the trial to minimize the risk of bias. All communication occurred between the research agency and the participants. Participants completed online self-report questionnaires hosted on a secure platform, UNICOM Intelligence (version 7.5.1; UNICOM Systems, Inc), at 3 time points: baseline (days 1-2), intervention testing phase (days 8-9), and 1 week after the intervention (days 15-16). Questionnaire functionality underwent user testing by authors NP, SH, and HW and research agency staff before the trial commenced.

At 8 AM CST on days 1 to 2 (time 1, baseline [T1]), days 8 to 9 (time 2, intervention testing phase [T2]), and days 15 to 16 (time 3 at 1 week after the intervention [T3]), the research agency sent participants a link to the corresponding questionnaire. Questionnaires delivered at T1 and T3 were composed of trait measures of body esteem, body appreciation, internalization of appearance ideals, and social media literacy. The T2 questionnaire was composed of state measures of body satisfaction, body functionality, and mood. The participants had 36 hours to complete each questionnaire and were instructed to do so in one sitting. The research agency assigned each participant their own unique participant identification number to match participant responses over time.

Following completion of the baseline questionnaire, participants were randomly allocated via the UNICOM Intelligence program using least filled quota to the intervention, active control, or attention control group, which was executed by the research agency. Concealing participants from their assigned group was not possible given the nature of the intervention; however, the agency was concealed from the participants’ randomized arm as the allocation was automated. One week later, on day 8 at 8 AM, participants were sent questionnaire 2 along with a link to play one of three games: (1) Super U Story (intervention group), (2) Rainbow Friends 2 Story (Color Story; active control group), or (3) an online word search (attention control group). The state measures were completed immediately before and after gameplay (ie, T2a and T2b). When participants completed the first set of state measures, they were instructed to play the game that corresponded to their allocated condition for a minimum of 5 minutes and no longer than 30 minutes based on a summary of preliminary gameplay analytics provided by Toya before executing the pilot (ie, raw numbers were not provided by Toya). Participants were instructed to complete the state measures and play the game in one sitting. Acceptability and game engagement information was also collected from the intervention group participants at the end of questionnaire 2. The final questionnaire (questionnaire 3) was sent to participants 1 week later, on day 15. In total, 3 attention checks were embedded within the T1 and T3 questionnaires to assess data quality, and 3 manipulation checks were presented at the end of the T2 survey to assess intervention and active control participants’ level of attention during gameplay. Participants were given 36 hours to complete each questionnaire. The research agency sent reminder messages to those who had not engaged with each questionnaire (after 8 hours for questionnaire 1 and after 8 and 26 hours for questionnaires 2 and 3, respectively). Multimedia Appendices 2-4 provide the questionnaires at each time point. Following the completion of questionnaire 3, the research agency sent participants a debriefing document disclosing the study’s aims and objectives and contacts for free mental health resources. The procedure outlined previously was first trialed in a pilot with 136 participants (see the Pilot Study subheading in the Results section).

### Measures

#### Primary Outcome Measure

The primary outcome measure was state-based body satisfaction using visual analogue scales (VASs) [66,67] immediately before and after gameplay. The three items were as follows: (1) “How happy do you feel about your body weight, right now?” (2) “How happy do you feel about your body shape, right now?” (3) “How happy do you feel about the way you look, right now?” Participants indicated their level of satisfaction on an 11-point VAS (0=*extremely dissatisfied*; 10=*extremely satisfied*). A mean score across the 3 items was calculated, with higher scores indicating higher state body satisfaction. VASs have been used widely among young people and been shown to be reliable and valid [31,68]. Internal consistency was high (Cronbach α=0.904; McDonald ω=0.912).

#### Secondary Outcome Measures

A total of 6 secondary outcome measures were included. An 11-point VAS was used to measure state mood using a single item (0=*very sad*; 10=*very happy*). Higher scores indicate a more positive mood. An 11-point VAS with the same anchors was also used to measure body functionality using a single item (“How happy do you feel with what your body can do right now?”) [69]. Higher scores indicate higher levels of body functionality. Trait body esteem was measured using the 20-item Body Esteem Scale for children [70], which has shown good reliability and validity with young people [71]. Response options were presented on a Likert-type scale from 1 (*never*) to 5 (*always*). Higher scores indicate higher body esteem. Internal consistency was high (Cronbach α=0.930; McDonald ω=0.930). The 10-item Body Appreciation Scale–2 for Children, validated with children aged 9 to 11 years, was used to assess trait body appreciation [72] on a Likert-type scale from 1 (*never*) to 5 (*always*). Higher scores indicate greater body appreciation. Internal consistency was high (Cronbach α=0.909; McDonald ω=0.910). The 12-item Internalization—general subscale of the Sociocultural Attitudes Toward Appearance Questionnaire [73] was also used to measure trait internalization of appearance ideals. This validated measure has been used widely across various populations and cultures, and it has been found to have strong reliability and validity [73-76]. Higher scores indicate greater internalization of appearance ideals. Internal consistency was high (Cronbach α=0.964; McDonald ω=0.963). A purpose-built measure assessed social media literacy via 3 single items using an 11-point VAS ranging from 0 (*totally disagree*) to 10 (*totally agree*), with higher scores indicating higher social media literacy. The items were as follows: (1) “When I post on social media, it’s important to focus on what I’m doing, not what I look like” (2) “I would know what to do if I was being teased or bullied about my appearance on social media” and (3) “It is important to think before I accept everything I see on social media is true.”

#### Intervention Acceptability

At the end of the T2 questionnaire, intervention participants were asked to respond to 3 open-ended questions regarding what they liked, disliked, and learned from the game. In addition, participants completed 4 self-report questions regarding intervention acceptability (ie, the extent to which they enjoyed the game, liked the story, liked the characters, and whether they would recommend the game). Responses ranged from 1 (*strongly disagree*) to 5 (*strongly agree*) on a Likert-type scale.

#### Engagement

Intervention engagement was assessed via 2 metrics: average time spent playing the game and self-reported engagement. Participants in the intervention condition responded to 8 questions that captured the key messages and activities they engaged with while playing Super U Story. These 8 questions provided a measure of intervention engagement. In total, 7 of the questions (eg, “Before traveling to the Academy, did you interact with the Selfie Guy?”) included response options of *yes*, *no*, or *not sure*. Each question was paired with a screenshot from Super U Story that reflected the question’s content but did not include any key messaging. The eighth question addressed engagement with the 25 pop-up messages via Super U Story’s social media site Flutter. Participants were asked how many Flutter messages they remembered reading; they were provided with the following response options: *0*, *1*, *2-3*, *4-6*, *7-10*, *11-15*, *16-20*, *more than 21*, and *not sure*.

### Sample Size

For this 3-arm study, based on an a priori sample size calculation, we aimed to recruit 322 participants per arm with complete data (N=966 in total) to have 80% power to detect a small standardized effect (Cohen δ=0.2) between the intervention and each control group using analysis of covariance (ANCOVA) conservatively assuming a correlation between baseline and outcome of at least 0.6. To account for dropout (similar studies testing microinterventions online show dropout rates of 34% at posttest [30]), phantom applications (ie, bots), and those who expressed an interest but did not fully engage (ie, manipulation check failures), the sample size was inflated by 35% to 493 per group (N=1479) to ensure sufficient power at posttest.

### Analyses

#### Acceptability Analysis

Qualitative feedback from girls and boys was analyzed using inductive content analysis [77]. Data were coded by the fifth author (MB), who was blinded to participant details. Codes were generated during the analytical process (ie, not using a predetermined codebook). The coder familiarized herself with the dataset by reading and rereading the responses. Author MB had routine check-ins with the second author (SH) to situate responses within the context of the trial and clarify any details during the coding process, such as confirming game elements that contained key messaging and streamlining and collapsing similar codes. The dataset was color coded and shared with SH, who reviewed all the codes. Any discrepancies or points of contention were resolved via discussion. The findings are the result of an iterative process of coding and recoding to ensure that the responses were accurately captured and shared. Quantitative feedback from girls and boys is reported as frequencies.

#### Engagement Analyses

Gameplay time was restricted to 30 minutes maximum. The first metric of intervention gameplay was calculated through time stamps in the datafile, which was the difference between the last pre-exposure state measure completed and the first postexposure state measure completed. The second metric to assess intervention engagement was self-report questions at the end of the T2 questionnaire just before the acceptability questions.

### Hypothesis Testing

Analyses were conducted using SPSS (version 29.0; IBM Corp). Condition allocation was concealed from the data analyst throughout data preparation and hypothesis testing to avoid interpretation bias.

This real-world remote-based online gaming intervention expectedly had some technical challenges, which could not be resolved in real time despite best efforts. For these reasons, the analyses were conducted per protocol (PP) rather than being intention-to-treat (ITT) analyses (ie, eligible consenting randomized participants who did not exercise the right to withdraw and had no known protocol deviations were included in the analysis). The corresponding ITT analyses are provided as supplementary materials and are commented on in the Discussion section.

For hypothesis 1 (state-based measures), the primary analysis of state body satisfaction after the intervention (T2b) used ANCOVA with state body satisfaction before the intervention (T2a) as the covariate and randomized arm as the independent variable. Underpinning model assumptions, including the homogeneity of regression line assumption, were assessed, and appropriate simplification was undertaken if the parallel line assumption was justifiable. The η_p_^2^ summarized effect size in the ANCOVA model. A preplanned repeated-measure ANCOVA was used to compare the intervention group against the active control group at T2b, and the same preplanned ANCOVA model was used to compare the intervention group against the attention control group. The same analysis plan was used for state body functionality and mood.

For hypothesis 2, the same ANCOVA strategy used for analyzing the state measures was used for the trait measures. Specifically, for each trait measure (ie, body esteem, body appreciation, internalization of appearance ideals, and social media literacy), ANCOVA was used for between-group comparisons at T3 after controlling for the commensurate baseline measure at T1.

Hypothesis 3 (moderation of effects by age group and gender) was similarly considered by extending the ANCOVA models. Specifically for gender, the ANCOVA model was extended to include a main effect for gender and a gender by group interaction term. Age was dichotomized (≤11 years and ≥12 years) and included in the ANCOVA modeling as a main effect for age and the age by group interaction term. Moderation was assessed using the interaction terms.

For hypothesis 4 (engagement), we planned to use regression analysis and consider the degree of correlation between measures of engagement (ie, length of time of gameplay and number of Flutter messages recalled) in the intervention arm and for each outcome measure (T2b and T3) after controlling for the commensurate measure at T1 or T2a.

## Results

### Pilot Study

#### Sample Size

For the external pilot study, we aimed to recruit a minimum of 30 participants per arm (total sample: N=90). As per recommendations by Whitehead et al [78], our target sample size was in line with that for a main trial designed with 90% power and 2-sided 5% significance; a minimum of 25 participants per arm were required to detect standardized small effect sizes (0.2), which align with the effects detected for similar web-based microinterventions [23,28,32].

The pilot study (N=136) was conducted in 2023 between January 7 and February 5. Participants’ ages ranged between 9 and 13 years (mean age 10.90, SD 1.36 years), and gender was evenly split between girls and boys (68/136, 50% girls). Participants resided in the 4 main regions of the United States: the Midwest (33/136, 24.3%), the Northeast (20/136, 14.7%), the South (58/136, 42.6%), and the West (25/136, 18.4%). Most participants (99/136, 72.8%) identified their ethnicity as White; the remaining participants identified as African American or Black (19/136, 14%), Asian (3/136, 2.2%), mixed (3/136, 2.2%), and other (7/136, 5.1%). The pilot study was conducted before the commencement of the main trial to assess (1) the quality of the data collected, (2) indications of harm (eg, negative change in body satisfaction in intervention participants), (3) the recruitment strategy, (4) participant retention across time points, (5) the acceptability of Super U Story, and (6) intervention engagement.

#### Pilot Study Results

Data quality was considered strong as 98.5% (134/136) of the participants correctly responded to the attention checks embedded in the measures at T1 and T3. Manipulation checks presented at the end of the T2 questionnaire for intervention participants showed that approximately half the intervention participants were paying attention during gameplay—57% (26/46), 54% (25/46), and 50% (23/46) of the participants provided correct responses to these 3 questions. Mean values were all within the expected range. No indication of harm was observed across the outcome measures for the intervention group, and no participants requested sources of support. The recruitment strategy was deemed successful (ie, the agency reached the target sample size within the stipulated time frame), and attrition was typical of rates observed for other eHealth interventions [79] from recruitment to T1 and across time points, particularly at T1 and T2. A total of 484 participants were recruited into the pilot; however, attrition between recruitment and starting the survey at T1 was 34.7% (168/484). Attrition rates across time points were as follows: 32.9% (104/316), 21.7% (41/189), and 2.2% (3/136) at T1, T2, and T3, respectively.

Acceptability of Super U Story was good, assessed via 4 statements measured on a 5-point Likert scale ranging from *totally disagree* to *totally agree*. The combined responses of *mostly agree* and *totally agree* showed that 80% (37/46) of the participants enjoyed playing the game, 83% (38/46) liked the story, 87% (40/46) liked the game’s characters, and 76% (35/46) indicated that they would recommend Super U Story to their friends.

The first metric to assess intervention engagement was the average time spent playing the game, which showed that gameplay (ie, Super U Story, Rainbow Friends 2 Story [Color Story], and the web-based word search) was fairly equal across conditions. More than half of the participants in each group played their assigned game for >20 minutes as follows: 59% (27/46) of intervention participants, 57% (24/42) of active control participants, and 58% (28/48) of attention control participants. The other metric consisted of responses from intervention participants regarding their engagement with 8 nonmandatory features of the game that included key messaging (Table 1). Overall, pilot participants’ engagement with Super U Story (46/136, 33.8%) was considered good by Roblox standards; according to Toya, engagement by pilot intervention participants was in line with or better than the engagement data with Super U Story collected in October 2022.

**Table 1:** Participants’ self-reported engagement with the intervention’s nonmandatory key messaging features in the pilot study (N=46).

Nonmandatory game feature	Participants, n (%)
Reading ≥11 Flutter messages	4 (9)
Reading the news on the bus station digital screens	14 (30)
Interaction with Selfie Guy in the lobby	31 (67)
Interaction with Academy lobby screens	21 (46)
Elevator conversations between nonplayer characters	31 (67)
Trainer interactions	24 (52)
Interaction with meditation mats	20 (43)
Reaching the end of the game	15 (33)

#### Study Design Changes Based on Pilot Study Results

Following the pilot, various changes were made to the study design. To increase response rates during the main trial, questionnaire reminders were sent via SMS text message and email, the window of time to complete each questionnaire was increased from 24 to 36 hours, key parts of the instructions were simplified, and participants who completed all 3 questionnaires were entered into a sweepstake run by the research agency to win 1 of 3 US $1000 prizes (Visa gift card claim codes) in addition to the US $40 incentive. In addition, 3 manipulation checks were added to the end of the T2 questionnaire for active control participants to gauge their level of attention during gameplay as with the intervention participants, and the response options for the social media literacy questions were changed from a Likert scale to a VAS to increase sensitivity. Finally, given that only a third of the participants reached the end of the game (15/46, 33%; Table 1), the Flutter messages containing key messaging were reordered so that most of them were presented earlier in the game. No further changes were made to the study design.

Regarding the intervention, edits to the text within Super U Story were implemented to strengthen the key messages. For example, in a scene in which players explore a dining hall that has laid out various types of food, the original text that was displayed when the player clicked on a plate—“This is it! Yummy! Just what my body wanted!”—was updated to “YUM!! I love giving my body what it needs instead of focusing on what it looks like.” In addition, some messages in the fictional social media platform Flutter that did not contain key messages were replaced with psychoeducational content. For example, one Flutter message that read the following—“Everyone’s so excited to see me at the Academy! What a loving welcome”—was changed to “Being bullied about your looks? Tell someone, ignore it, or block them. Follow people who make you feel good!!!!”

### Main Trial

#### Overview

**Figure 2:**
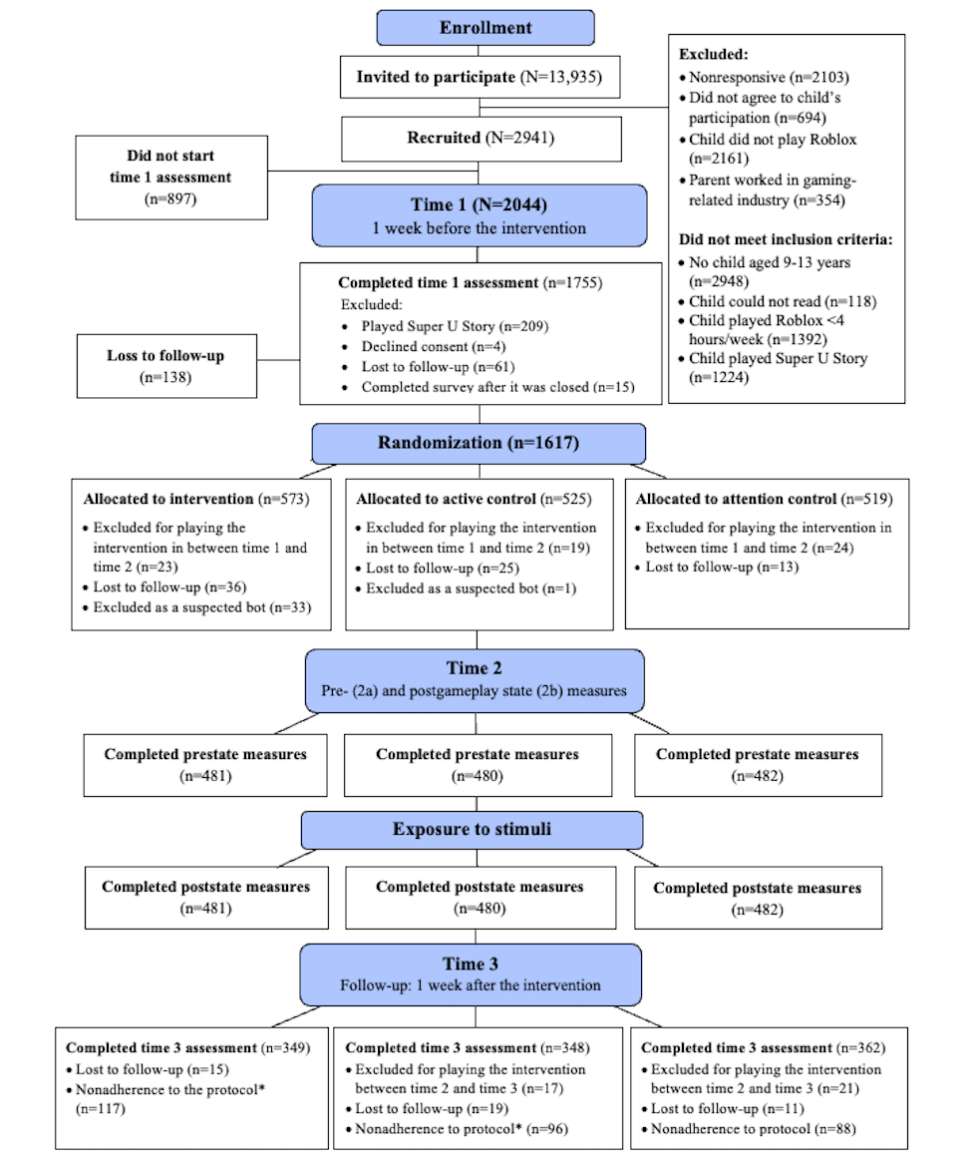
Research design and participant flow using the CONSORT-EHEALTH (Consolidated Standards of Reporting Trials of Electronic and Mobile Health Applications and Online Telehealth) guidelines. *Protocol nonadherence included any of the following: participants who played their assigned game for <5 minutes, reported technical difficulties, reported problems returning to the survey following gameplay, or answered at least one manipulation check incorrectly (intervention and active control only).

Recruitment was conducted between February 20, 2023, and March 3, 2023, and the trial was conducted between March 4, 2023, and March 19, 2023. The participant flow diagram is shown in Figure 2. In total, 1059 children and adolescents aged between 9 and 13 years (mean age 10.91, SD 1.36 years) participated. The sample involved a similar number of girls (460/1059, 43.4%) and boys (599/1059, 56.6%). Most participants (761/1059, 71.9%) identified their ethnicity as White. Just over half (549/1059, 51.8%) of the participants belonged to the midrange socioeconomic group. A third of the participants (358/1059, 33.8%) resided in the South of the United States; the remaining participants came from the other 4 regions, with the fewest participants from the Northeast (197/1059, 18.6%). Just over two-thirds of the participants (703/1059, 66.4%) played Roblox games an average of 4 to 10 hours a week. Table 2 provides the complete baseline demographic data.

**Table 2:** Participant baseline demographic data (N=1059)^a^.

Variable		Total sample	Intervention (n=349)	Active control (n=348)	Attention control (n=362)	*P* value	
Age (y), mean (SD)		10.91 (1.36)	10.93 (1.32)	11.00 (1.39)	10.81 (1.37)	.20	
**Age (y), n (%)**							—^b^
	9	208 (19.6)	63 (18.1)	62 (17.8)	83 (22.9)		
	10	234 (22.1)	78 (22.3)	79 (22.7)	77 (21.3)		
	11	230 (21.7)	81 (23.2)	72 (20.7)	77 (21.3)		
	12	216 (20.4)	76 (21.8)	67 (19.3)	73 (20.2)		
	13	171 (16.1)	51 (14.6)	68 (19.5)	52 (14.4)		
**Gender, n (%)**							.10
	Girls	460 (43.4)	154 (44.1)	136 (39.1)	170 (47)		
	Boys	599 (56.6)	195 (55.9)	212 (60.9)	192 (53)		
**Ethnicity, n (%)**							.28
	American Indian or Alaska Native	11 (1)	5 (1.4)	2 (0.6)	4 (1.1)		
	Asian	40 (3.8)	11 (3.2)	9 (2.6)	20 (5.5)		
	Black or African American	111 (10.5)	34 (9.7)	40 (11.5)	37 (10.2)		
	Hispanic	133 (12.6)	38 (10.9)	47 (13.5)	48 (13.3)		
	Native Hawaiian or Pacific Islander	2 (0.2)	0 (0)	1 (0.3)	1 (0.3)		
	White	761 (71.9)	261 (74.8)	249 (71.6)	251 (69.3)		
	Other	1 (0.1)	0 (0)	0 (0)	1 (0.3)		
**Socioeconomic status, n (%)**							.69
	Low	228 (21.5)	76 (21.8)	68 (19.5)	84 (23.2)		
	Middle	549 (51.8)	180 (51.6)	186 (53.4)	183 (50.6)		
	High	282 (26.6)	93 (26.6)	94 (27)	95 (26.2)		
**Region, n (%)**							.37
	Midwest	214 (20.2)	73 (20.9)	74 (21.3)	67 (18.5)		
	Northeast	197 (18.6)	58 (16.6)	75 (21.6)	64 (17.7)		
	South	358 (33.8)	126 (36.1)	102 (29.3)	130 (35.9)		
	West	290 (27.4)	92 (26.4)	97 (27.9)	101 (27.9)		
**Average Roblox play per week (h), n (%)**							.34
	4-10	703 (66.4)	240 (68.8)	225 (64.7)	238 (65.7)		
	11-20	298 (28.1)	92 (26.4)	97 (27.9)	109 (30.1)		
	21-30	43 (4.1)	14 (4)	19 (5.5)	10 (2.8)		
	31-40	13 (1.2)	3 (0.9)	5 (1.4)	5 (1.4)		
	>40	2 (0.2)	0 (0)	2 (0.6)	0 (0)		

^a^Test statistic: 1-way between-subject ANOVA for age and chi-square test of association for all other demographic variables.

^b^Not applicable.

#### Baseline Characteristics

There were no differences in any demographic variables between the randomized groups (Table 2). ANOVA showed that means before the intervention (ie, T1 and T2a) did not significantly differ between randomized groups for body satisfaction (*P*=.58), mood (*P*=.91), body functionality (*P*=.42), body esteem (*P*=.06), internalization (*P*=.06), or the social media literacy items (*P*=.57, *P*=.71, and *P*=.37). Baseline mean differences at T1 were observed for body appreciation (*P*=.02). Post hoc analyses using the Fisher least significant difference test [80] indicated that the mean for the intervention group was significantly higher than that for the active control group (*P*=.02; *d*=0.185) and mean body appreciation was significantly higher in the attention control group than in the active control group (*P*=.02; *d*=0.175) but with no significant difference in mean body appreciation between the intervention and attention control groups (*P*=.93). These differences were controlled for in the analyses. Means and SDs for all measures are provided in Table 3.

**Table 3:** Means and SDs for the measures at each time point.

Measure and time point		Intervention (n=349)		Active control (n=348)		Attention control (n=362)	
		Participants, n (%)	Mean (SD)	Participants, n (%)	Mean (SD)	Participants, n (%)	Mean (SD)
**Body satisfaction**							
	T2a^a^	349 (100)	7.980 (1.7417)	348 (100)	7.924 (1.7936)	362 (100)	8.061 (1.7474)
	T2b^b^	349 (100)	8.315 (1.4703)	348 (100)	8.134 (1.7405)	362 (100)	8.292 (1.7702)
**Mood**							
	T2a	349 (100)	8.170 (1.6236)	348 (100)	8.213 (1.4394)	362 (100)	8.217 (1.5754)
	T2b	349 (100)	8.498 (1.5020)	348 (100)	8.586 (1.3388)	362 (100)	8.344 (1.7303)
**Body functionality**							
	T2a	349 (100)	8.205 (1.7394)	348 (100)	8.236 (1.5999)	362 (100)	8.360 (1.6450)
	T2b	349 (100)	8.417 (1.6142)	348 (100)	8.421 (1.5570)	362 (100)	8.438 (1.6226)
**Body esteem**							
	T1^c^	349 (100)	3.754 (0.6999)	348 (100)	3.660 (0.7216)	362 (100)	3.780 (0.6995)
	T3^d^	343 (98.3)	3.767 (0.7421)	323 (92.8)	3.746 (0.7542)	341 (94.2)	3.771 (0.7653)
**Body appreciation**							
	T1	349 (100)	4.022 (0.6782)	348 (100)	3.893 (0.7078)	362 (100)	4.017 (0.7042)
	T3	343 (98.3)	4.042 (0.6645)	323 (92.8)	4.041 (0.6719)	341 (94.2)	4.067 (0.7162)
**Internalization**							
	T1	349 (100)	2.655 (1.1290)	348 (100)	2.825 (1.1489)	362 (100)	2.645 (1.1224)
	T3	343 (98.3)	2.683 (1.1807)	323 (92.8)	2.827 (1.1951)	341 (94.2)	2.712 (1.1737)
**Social media literacy: item 1**							
	T1	349 (100)	7.158 (2.6130)	348 (100)	6.961 (2.7434)	362 (100)	7.123 (2.4990)
	T3	342 (98)	7.705 (2.1604)	323 (92.8)	7.633 (2.2206)	341 (94.2)	7.748 (2.4098)
**Social media literacy: item 2**							
	T1	349 (100)	7.364 (2.4624)	348 (100)	7.209 (2.5053)	362 (100)	7.291 (2.5075)
	T3	343 (98.3)	7.958 (2.0864)	323 (92.8)	7.758 (2.1055)	341 (94.2)	7.989 (2.0543)
**Social media literacy: item 3**							
	T1	348 (99.7)	8.358 (1.8336)	348 (100)	8.251 (2.0277)	362 (100)	8.451 (1.7869)
	T3	343 (98.3)	8.687 (1.4951)	322 (92.5)	8.414 (1.9664)	341 (94.2)	8.622 (1.7341)

^a^Time point for data collection of state measures immediately before gameplay.

^b^Time point for data collection of state measures immediately after gameplay.

^c^Time point for data collection of trait measures 1 week before the intervention.

^d^Time point for data collection of trait measures 1 week after the intervention.

#### Missing Data

Missing data in this study were minimal. At each of T1, T2a, and T2b, only 0.1% (1/1059) of the participants failed to provide data. At T3, a total of 5% (53/1059) failed to provide outcome data. Dropout between T1 and T2 was not related to age (*P*=.14), gender (*P*=.53), region (*P*=.53), or urbanicity (*P*=.88). However, 19.9% (274/1379) of those from the low or middle socioeconomic groups dropped out between T1 and T2 compared with 9.9% (37/375) of those with a high socioeconomic status (*P*<.001). Mean body esteem (*P*=.20), body appreciation (*P*=.10), internalization (*P*=.41), and social media literacy (*P*=.29, *P*=.17, and *P*=.53) scores at T1 did not significantly differ among those who dropped out at T2.* *At T3, the dropout rate was 1.7% (6/349) in the intervention group, 7.2% (25/348) in the active control group, and 5.8% (21/362) in the attention control group. In general, these observed levels of missingness are considered not sufficiently large to be of major concern [81,82]. Missingness was not gender dependent (*P*=.40) or dependent on age group (*P*=.69). Those with missing data at T3 did not systematically differ from complete responders at T1 on the 3 social media literacy items (*P*=.56, *P*=.15, and *P*=.13) or on body esteem (*P*=.71) or body appreciation (*P*=.58). Multiple imputation for the small amount of missing data was undertaken. The resulting statistical analyses yielded the same statistical conclusions irrespective of whether the small amount of imputed data was accounted for except for 1 instance in which a nonsignificant result without imputation was significant after imputation. In this conflicting situation, the mean of the third social media literacy item was significantly higher in the active control group than in the intervention group (*P*=.03) but with a small effect. For these reasons, we report results without imputation.

#### Intervention Acceptability

Most participants enjoyed playing the game (269/349, 77.1%), liked the story (268/349, 76.8%), liked the game’s characters (276/349, 79.1%), and would recommend the game to their friends (238/348, 68.4%). Regarding the qualitative acceptability data, findings from the content analysis can be found in Multimedia Appendix 5. In total, 349 participants provided 361 responses describing what they learned. In total, 29.1% (105/361) of the responses reported learnings related to the key messaging in the intervention, such as learning about body functionality and appreciation (23/361, 6.4%) and social media literacy (10/361, 2.8%) and that everybody is unique (20/361, 5.5%), among other learnings. Additional learnings included how to be creative (9/361, 2.5%) and persevere (11/361, 3%). Apart from this, participants responded with learnings that did not correspond to key intervention messaging (66/361, 18.3%) or did not answer the question asked (ie, miscellaneous responses; 190/361, 52.6%). When asked what they liked about the game, 348 participants provided 365 responses. In total, 12.1% (44/365) of the responses were related to key messaging, with participants specifically reporting that they liked the positive messages (37/365, 10.1%). Other popular responses included liking the characters (66/365, 18.1%) and the superhero aspects (44/365, 12.1%), among others. In response to the final question, 349 participants provided 356 responses describing what they disliked about the game. A large proportion of the responses (158/356, 44.4%) reported that there was nothing that the participants did not like. Dislikes related to talking about body image (9/356, 2.5%), and a small fraction felt that the game was for girls (2/356, 0.6%). Other dislikes included finding the game boring (15/356, 4.2%) and issues related to the mechanics of the game, such as moving around (63/356, 17.7%). A breakdown of the complete content analysis with example responses and all response categories is available in Multimedia Appendix 5.

#### Engagement

Median gameplay time for the intervention group (15.2, IQR 10.6-21.2 min), active control group (15.4, IQR 10.3-21.7 min), and attention control group (13.1, IQR 9.1-19.6 min) showed some variation (*P*=.04), with no significant difference between the intervention group and active control group (*P*=.72) but with a shorter game duration for the attention control group than for the intervention group (*P*=.046) and the active control group (*P*=.02).

Self-reported engagement with the nonmandatory game features that contained key messages can be found in Table 4. The most interacted with element was Selfie Guy in the lobby early in the game, followed by conversations between nonplayer characters.

**Table 4:** Participants’ self-reported engagement with the 8 nonmandatory key messaging features of the intervention in the main trial (N=349).

Nonmandatory game feature	Participants, n (%)
Reading ≥11 Flutter messages	36 (10.3)
Reading the news on the bus station digital screens	120 (34.4)
Interaction with Selfie Guy in the lobby	239 (68.5)
Interaction with Academy lobby screens	165 (47.3)
Elevator conversations between nonplayer characters	233 (66.8)
Trainer interactions	184 (52.7)
Interaction with meditation mats	116 (33.2)
Reaching the end of the game	67 (19.2)

#### Main Statistical Analyses

#### Overview

The parallel line assumption in the ANCOVA models was considered justified, and as such, we reported the main effect of whether randomized arms differed after controlling for baseline.

Table 5 summarizes the results of the preplanned ANCOVA for the omnibus 3-group comparison and the preplanned comparisons between the intervention group and the active control group and between the intervention group and the attention control group.

**Table 5:** Results of the preplanned analysis of covariance for the omnibus 3-group comparison and the preplanned comparisons between the intervention group and the active control group and between the intervention group and the attention control group.

Measure and comparison			*F* test (*df*)	*P* value	η^2^
**Time 2a^a^ vs time 2b^b^**					
	**Body satisfaction**				
		Omnibus	2.514 (2, 1055)	.08	0.005
		IG^c^ vs ACG^d^	5.196 (1, 694)	.02	0.007
		IG vs AttCG^e^	2.258 (1, 708)	.13	0.003
	**Mood**				
		Omnibus	3.917 (2, 1055)	.02	0.007
		IG vs ACG	0.556 (1, 694)	.46	0.001
		IG vs AttCG	3.664 (1, 708)	.06	0.005
	**Body functionality**				
		Omnibus	0.865 (2, 1055)	.42	0.002
		IG vs ACG	0.055 (1, 694)	.82	0.000
		IG vs AttCG	1.427 (1, 708)	.23	0.002
**Time 1^f^ vs time 3^g^**					
	**Body esteem**				
		Omnibus	4.843 (2, 1003)	.008	0.010
		IG vs ACG	5.398 (1, 663)	.02	0.008
		IG vs AttCG	0.361 (1, 681)	.55	0.001
	**Body appreciation**				
		Omnibus	3.610 (2, 1003)	.03	0.007
		IG vs ACG	6.078 (1, 663)	.01	0.009
		IG vs AttCG	0.833 (1, 681)	.36	0.001
	**Internalization**				
		Omnibus	1.671 (2, 1003)	.19	0.003
		IG vs ACG	1.427 (1, 663)	.23	0.002
		IG vs AttCG	0.376 (1, 681)	.54	0.001
	**Social media literacy: item 1**				
		Omnibus	0.122 (2, 1002)	.89	0.000
		IG vs ACG	0.051 (1, 662)	.82	0.000
		IG vs AttCG	0.263 (1, 680)	.61	0.000
	**Social media literacy: item 2**				
		Omnibus	1.057 (2, 1003)	.35	0.002
		IG vs ACG	1.207 (1, 663)	.27	0.002
		IG vs AttCG	0.081 (1, 681)	.78	0.000
	**Social media literacy: item 3**				
		Omnibus	1.708 (2, 1001)	.18	0.003
		IG vs ACG	3.369 (1, 661)	.07	0.005
		IG vs AttCG	0.588 (1, 680)	.44	0.001

^a^Time point for data collection of state measures immediately before gameplay.

^b^Time point for data collection of state measures immediately after gameplay.

^c^IG: intervention group.

^d^ACG: active control group.

^e^AttCG: attention control group.

^f^Time point for data collection of trait measures 1 preintervention.

^g^Time point for data collection of trait measures 1 week postintervention.

#### Hypothesis 1

Hypothesis 1 tested whether playing Super U Story produced immediate increases in state body satisfaction, mood, and body functionality. For the primary outcome of body satisfaction, the omnibus repeated-measure ANCOVA indicated that means immediately after the intervention (T2b) did not significantly differ between groups (*F*_2, 1055_=2.514; *P*=.08; η_p_^2^=0.005). Mean body satisfaction was significantly higher in the intervention group than in the active control group (*P*=.02) but mean body satisfaction was not higher in the intervention and the attention control group (*P*=.13). For state mood, there was a between-group effect at T2b controlling for baseline (*F*_2, 1055_=3.917; *P*=.02; η_p_^2^=0.007), but the comparison between the intervention and the active control groups did not achieve statistical significance (*P*=.46), nor did the comparison between the intervention and the attention control groups (*P*=.06). At T2b, there was no significant between-group difference for body functionality (*F*_2, 1055_=0.865; *P*=.42; η_p_^2^=0.002); comparisons between the intervention and the active control groups (*P*=.82) and between the intervention and the attention control groups (*P*=.23) were not statistically significant.

#### Hypothesis 2

Hypothesis 2 tested the differences in the trait outcomes of body esteem, body appreciation, internalization of appearance ideals, and social media literacy at 1 week after the intervention (T3). At T3, mean body esteem significantly varied between randomized arms (*F*_2, 1003_=4.843; *P*=.008) after controlling for baseline. Mean body esteem was significantly lower in the intervention group than in the active control group (*F*_1, 663_=5.398; *P*=.02) but was not significantly different from that in the attention control group (*F*_1, 681_=0.361; *P*=.55). Similarly, at T3, mean body appreciation significantly varied between at least 2 randomized arms (*F*_2, 1003_=3.610; *P*=.03), with mean body appreciation being significantly lower in the intervention group than in the active control group (*F*_1, 663_=6.078; *P*=.01), but there was no significant difference between the intervention and the attention control groups (*F*_1, 681_=0.833; *P*=.36). At T3, mean values for internalization (*P*=.19) and the 3 social media literacy items (*P*=.89, *P*=.35, and *P*=.18) were not significantly different between groups.

#### Hypothesis 3

Hypothesis 3 tested whether differences in state and trait outcomes at both time points (immediately after the intervention and 1 week after the intervention) were moderated by participant gender (girls or boys) and age group (9-11 years and 12-13 years). Participant gender and age group did not moderate any outcome variables. Specifically, any effects for body satisfaction were not moderated by gender (*P*=.39) or age group (*P*=.08). Similarly, gender (*P*=.63) and age group (*P*=.55) did not significantly moderate effects for state mood. Finally, for state body functionality, moderation effects for gender (*P*=.52) or age group (*P*=.58) were not statistically significant. In terms of the trait outcomes, gender (*P*=.56) and age group (*P*=.91) did not have moderating effects for body esteem. For body appreciation, there were no moderating effects of gender (*P*=.34) and age group (*P*=.77). Similarly, gender (*P*=.97) and age group (*P*=.77) did not have moderating effects for internalization. Finally, for the 3 social media literacy items, there were no moderating effects of gender (*P*=.94, *P*=.20, and *P*=.39) or age (*P*=.96, *P*=.70, and *P*=.77). Multimedia Appendix 6 provides the *P* values for the potential moderating effect of age and gender for all outcomes.

#### Hypothesis 4

The exploratory analysis set out to examine whether greater engagement with the key messaging and activities in the intervention condition would result in greater improvements in state- and trait-based outcomes. However, the quality of the engagement data hampered our ability to run reliable dose-response effects. Certain data points had a disproportionate influence on model fit, thereby impacting the results, deeming potential effects questionable, unreliable, and likely nonreplicable. As a result, we were unable to run the exploratory analysis as planned. The Limitations subheading in the Discussion section provides further details.

#### ITT Analyses

While the analyses used in the trial were PP, we have included a summary of the ITT analyses in Multimedia Appendix 7. Most contrasts and analyses yielded identical conclusions in both the ITT and PP analysis set. There was 1 contradictory finding in which the PP analysis showed a statistically significant effect for state body satisfaction whereas the ITT analysis did not. In this case, improvements in state body satisfaction immediately after playing Super U Story in comparison to the active control were no longer present. In addition, there were a small number of contrasts in which the ITT analysis set showed statistical significance but significance was not observed in the PP analysis set. These effects include a statistically significant difference in mean mood score between the Super U Story arm and the attention control arm (*P*=.009) and a significantly higher mean social media literacy score (on item 2 only) for Super U Story than for the active control (*P*=.01).

## Discussion

### Principal Findings

This 3-arm, fully-powered RCT evaluated the effectiveness of a purpose-built body image Roblox game, Super U Story, on children’s and adolescents’ state and trait body image and related outcomes. The findings partly support hypothesis 1—young people who played Super U Story experienced significant improvements in state body satisfaction immediately after playing Super U Story in comparison to participants in the active control group (Rainbow Friends 2 Story [Color Story]) but not when compared to the attention control group (word search). This effect was very small, in line with previous body image research using universal samples [20]. Unexpectedly, young people who played Super U Story did not experience improved state mood or state body functionality immediately after gameplay in comparison to the control groups. Moreover, contrary to hypothesis 2, intervention participants did not report improved trait body esteem, body appreciation, social media literacy, or reduced internalization of appearance ideals in comparison to the control groups at the 1-week follow-up. Body esteem and body appreciation were significantly lower among those who played Super U Story than among those who played Rainbow Friends 2 Story (Color Story; active control) at 1 week after gameplay. These effects were weak. Regarding hypothesis 3, the exploratory analyses found that effects were not moderated by age group or gender. These null findings are inconsistent with our hypothesis. Furthermore, we were unable to explore dose effects given the poor quality of the engagement data and, thus, were unable to test hypothesis 4.

Importantly, there was no evidence that Super U Story causes harm, meaning that, although there were no robust positive effects, we are confident that Super U Story does not negatively impact body image and related factors for girls and boys. On the basis of these findings, we conclude that Super U Story is ineffective in eliciting improvements in participants’ body image and related measures in comparison to both control conditions.

While the findings of this study were unexpected, they mirror unexpected findings in other body image intervention studies more broadly. Indeed, several micro- and standard intervention studies have also reported improvements in body image–related variables among the active control group [30,83,84]. Positive effects among the active control group have previously been attributed to demand or practice effects [84] or positive self-regard for participating in research [85], which may also be the case in this study. Relatedly, several studies have reported null findings for body image interventions [86-88], with a third of universal body image interventions found to be ineffective [89]. In their meta-analysis of stand-alone body image interventions, Alleva et al [13] noted that a considerable proportion of interventions that found negative or null effects for body image were not published or submitted for publication, limiting what can be learned from previous trials.

With these points in mind, we propose several possible explanations for why Super U Story did not have the expected impact on our key outcomes. First, the use of psychoeducation may have contributed to the nonsignificant findings. Key messaging was integrated into Super U Story (eg, via Flutter, the game’s social media platform) with information (eg, “Beware, some images on Flutter have been altered using editing tools. Don’t feel pressure to edit images of yourself, show the real you”) presented in a unidirectional way without opportunity for participants to engage in critical evaluation or practice new skills. A recent systematic review of digital interventions found that interactive interventions (ie, those that include additional building activities, such as written, listening, or reading activities or worksheets; assignments; and discussions) were most effective in improving body image outcomes among adolescents and young women [48]. In addition, another systematic review and meta-analysis looking at body image and media literacy interventions for young people found that interventions that induced and provided opportunities to resolve cognitive dissonance in participants were the most effective [90]. Research has shown that psychoeducational messaging is a weaker intervention strategy for changing behavior and attitudes (ie, body image) than other deeper learning activities in which participants have the opportunity to apply their knowledge. For instance, in a recent review, Guest et al [11] found limited evidence for psychoeducation as an intervention strategy for improving positive body image among children, with only 3 out of 9 psychoeducation-based interventions finding improvements across measures of body image. However, given the scope and nature of the Super U Story intervention (ie, brief, light touch, and fast-paced, with restrictions based on the platform used and software available), psychoeducation was chosen as an intervention strategy due to the ease of implementation in comparison to other strategies [11]. Furthermore, research has shown that this can be a useful approach in microinterventions; indeed, Matheson et al [30] found that mandatory psychoeducational messages delivered via microinterventions in both static and interactive formats were equally effective in immediately improving young people’s body image and mood in the short term.

Second, the intervention format may have contributed to the unexpected findings. Exposure to the key messaging via Super U Story was mostly optional given the typical conventions of Roblox games and the expectations of their users. The engagement data collected in this trial, albeit self-reported, provided some insight into how participants interacted with the game. The data suggest that participants experienced highly variable and often low-dose exposure. Notably, less than a third of the responses from participants about their learnings (105/361, 29.1%) related to the intervention’s key messaging. Previous successful microinterventions have included mandatory exposure to key messaging and activities [30]. In addition, player expectations and multiple distractions within the game may have contributed to the mixed findings. Players expect entertainment and escape from playing Roblox games. Overcoming obstacles (“obbies”) is central to that experience, but Super U Story’s obbies did not deliver key messaging and were likely a competing distraction. The Super U game developers were concerned about the “chocolate-covered broccoli” phenomenon (ie, losing players who object to thinly disguised educational messages [58,59]); as such, when writing the script for Super U Story, they were keen to avoid this risk by not enforcing mandatory exposure to the intervention’s psychoeducation messaging. However, because of this approach, the intervention’s messaging was likely to be insufficiently explicit to confer benefits, especially given the low levels of exposure.

Third, developmental factors in relation to processing educational content may have played a role. In this trial, the characters used in Super U Story were markedly different to the standard, blocklike characters typically featured in Roblox games, which was evident in some of the qualitative feedback we received. The storyline was also new to participants. According to the capacity model, how children process educational content is affected by their relatability to and familiarity with a narrative [91]. When narrative and educational content is less relatable and familiar, a higher cognitive load is required to process it. Participants in the Super U Story condition had no familiarity with the format of the characters and the storyline (inclusion criteria stated that participants must never have played Super U Story), which may have lessened the salience of the psychoeducational messages, consequently reducing the impact of the intervention. Conversely, the positive effects experienced by the active control group may be, in part, related to the familiarity of playing a game with the typical-looking Roblox characters. Going forward, given evidence that more familiar content reduces cognitive load [91], it would be useful to look at the impact of multiple engagements with Super U Story (ie, when participants are more familiar with the game). Moreover, microintervention studies, although with adult samples, have shown that multi-session and multi-activity approaches are effective at improving body image [23], further highlighting the importance of looking at the impact of repetitive gameplay.

Fourth, while the Super U Story acceptability scores were good, they have been typically higher for other similar interventions. For example, acceptability scores for the Brazilian body image chatbot Topity [32] and the Indonesian body image social media video series Warna-Warni Waktu [42] were generally of ≥85% (in comparison to the average acceptability score of 75.27%, [SD 4.83] for Super U Story). Coproduction (beyond user testing) and greater input from the target population (ie, those aged 9-13 years) at the earliest stages of development may have increased acceptability scores and potentially improved engagement levels. Indeed, evidence indicates that coproduction has been associated with improved engagement and adherence in other eHealth interventions [92,93].

### Limitations

This trial has several limitations. It was exclusively online (ie, participants completed all elements of the trial at home or in another non–laboratory-based setting), which was ecologically valid but, ultimately, limited the amount of control we had over the trial (eg, to troubleshoot problems), potentially impacting the quality of the data collected. Indeed, some participants indicated that they experienced glitches or struggled to return to the survey after playing the game, highlighting the challenges of collecting this type of data in a fully remote trial. We ensured that there was a “support” button embedded in the survey, which participants could access to receive help from a member of the research agency, but few chose to use this resource. A key reason for conducting the trial remotely was to enhance ecological validity (ie, participants were able to play the game in a familiar setting using their own device). The web-based design was also cost-effective and facilitated a more diverse geographic sample. However, it should also be noted that, in the review by Mahon and Seekis [48] of digital body image interventions, they found that tightly controlled, laboratory-based, and researcher-led studies tend to report significant results, whereas at-home settings result in more variable findings.

A further limitation was that we relied heavily on self-report estimates to measure engagement. In terms of capturing participants’ engagement with the game’s key features, we were unable to record this in a more objective way (ie, to track individual’s actual gameplay). Toya, the game developers, were able to collect engagement data on aggregate but were unable to do so for each individual player due to data protection regulations related to minors. Therefore, the quality of engagement data was unreliable (ie, participants relied on recall) and lacked sufficient detail. As a result, we were unable to conduct exploratory analyses to examine the impact of dose on body image and related outcomes. Future gaming trials would benefit from efficiently capturing engagement levels by tracking participants’ real-life gameplay to determine whether participants interact with any nonmandatory features (ie, receive the key messaging) [94] and calculate dose response. This is particularly noteworthy given that research suggests that self-reported adherence to digital interventions is inflated in comparison to objective adherence [94]. Crucially, obtaining fully objective adherence data (ie, not self-reported) could provide insights into whether engagement with key messaging was insufficient or the intervention was not potent enough to generate the desired effects [94]. Relatedly, in terms of metrics and engagement in the gaming world, the level of engagement evidenced in this study (albeit self-reported) was considered good. However, from an intervention perspective, it was very low, calling into question the fit of this game in improving children’s and adolescents’ body image.

An additional limitation was that baseline means across several of our variables were high, including social media literacy, mood, and body functionality, suggesting that many of our participants already had “good” levels before intervention exposure, consequently allowing for little room for improvement. It is possible that issues, for example, with mood and body functionality are less salient in this age group [12]. As such, future research with children could further explore these variables among younger and older cohorts to understand the optimal age for delivering such interventions.

Finally, this study used a PP analysis instead of an ITT analysis. While ITT is typically used for intervention trials, we opted for a PP approach due to concerns about the quality of the ITT data. Issues such as noncompliance, attention check failures, technical problems, and questionable data points (as detailed previously) led us to believe that the ITT analysis dataset contained data that were difficult to reconcile, whereas the PP analysis data had fewer unresolved queries. Therefore, we made the decision to report on the PP analysis dataset as clearly stated previously. While PP analyses are sometimes criticized for potentially showing more positive outcomes, our results from the PP analysis did not support the intervention’s efficacy. In fact, we concluded that the Super U Story intervention was not effective. For transparency, we have included the ITT results in Multimedia Appendix 7, and despite some differences, the overall conclusion that the intervention was not effective remains unchanged*.*

### Strengths

There are several strengths associated with this trial. It was adequately powered to detect small effects, and we included both an active control (alternative Roblox game) and an attention control (word search or non-Roblox game activity) to provide a fuller picture of the impact of Roblox games on body image and related factors. Using both types of control groups in a trial can help researchers understand and interpret the findings. Indeed, this study was developed at cost to have 2 control arms to permit a comprehensive assessment of efficacy. The prospective study has a preregistered primary research question, with a preregistered primary end point using a validated measure with good test-retest reliability. Sample size was determined a priori based both on power considerations and dropout rates informed by the literature; the target sample size was achieved. Analyses were conducted using well-established statistical techniques with due regard to statistical model assumptions to help ensure rigor, rely on statistical conclusions, and limit the impact of type I errors.

This study was also innovative in that we embedded a body image microintervention into a hugely popular gaming platform with a massive reach. Relatedly, we had the opportunity to inform experienced game developers as they created the body image game for Roblox. We were able to draw on their expert insight into game design and gameplay and understand what was feasible in terms of game development given the software available at the time. However, working with the developers involved compromise. Our priority as researchers was to maximize participant exposure to core messaging. In contrast, the game developers were opposed to including too much mandatory psychoeducational content for fear of losing players as, understandably, their business is to retain players until the end of the game. Finally, a notable strength of this study is the importance of sharing our learnings with the scientific community and not contributing to the file drawer problem or publication bias, in which studies without significant effects are not published [95]. Specifically, our detailed reporting of this study’s null effects contributes to improving the reliability of the wider evidence base [96] of intervention studies aiming to improve body image.

### Conclusions

This is the first large-scale RCT assessing a body image intervention, Super U Story, embedded within a popular gaming platform, Roblox. Overall, playing Super U Story did not cause harm; however, there is a lack of evidence to suggest that Super U Story improves body image or related factors. Despite the lack of effects, this trial demonstrated that, with stakeholder collaboration, there is scope to explore delivering microinterventions to young people via large commercial gaming platforms to help address the need to reach young people at scale.

## Appendix

Multimedia Appendix 1 Summary of the Super U Story intervention.

Multimedia Appendix 2 Time 1 questionnaire.

Multimedia Appendix 3 Time 2 questionnaire.

Multimedia Appendix 4 Time 3 questionnaire.

Multimedia Appendix 5 Content analysis of qualitative data.

Multimedia Appendix 6 *P* values for the moderating effects of age and gender.

Multimedia Appendix 7 Intention-to-treat analyses.

Multimedia Appendix 8 CONSORT eHEALTH checklist (V 1.6.1).

## Abbreviations

ANCOVA: analysis of covariance
ITT: intention-to-treat
PP: per protocol
RCT: randomized controlled trial
VAS: visual analogue scale

## Data Availability

The datasets generated or analyzed during this study are available from the corresponding author on reasonable request.
