# Supplementary material for: Evaluating the Effectiveness of a Roblox Video Game (Super U Story) in Improving Body Image Among Children and Adolescents in the United States: Randomized Controlled Trial
Source: J Med Internet Res. 2025 Jul 31;27:e66625. doi: 10.2196/66625 (PMC12355144; doi:10.2196/66625)
Supplement: Multimedia Appendix 1 [file jmir_v27i1e66625_app1.pdf]

Multimedia Appendix: Summary of the Super U Story intervention.

Activity		Explanation	Example	Social media literacy	Appearance comparisons	Body functionality	Body appreciation	Internalization of appearance ideals	Mandatory
1	Flutter messages (designed to mimic social media posts)	15 out of 25 pop-up messages from a fictional social media platform "Flutter" contain key messaging.	"You'll succeed in the Academy if you focus on what your body can do, NOT what it looks like. It'll also BOOST your confidence!"	✓	✓	✓	✓	✕	✓[a] ✕[b]
2	Digital news	3 monitors featuring "Self Esteem News" in the bus station lobby.	"New study finds 1 in 2 girls say unrealistic beauty advice on Flutter causes low self-esteem: Don't believe everything you see, researchers tell us."	✓	✓	✕	✕	✕	✕
3	Selfie Guy	Dialogue between the player and a non-player character, who is taking selfies in The Academy lobby.	Player: "Do you have to use a filter?"<br><br>Selfie Guy: "Um, duh. Everyone uses filters on everything. When you look perfect, that's when you get likes! …. Wait. That's messed up, isn't it? Ugh. I need to think before I post"	✓	✕	✕	✕	✕	✕

4	Computers	In The Academy lobby, three monitors feature 4 questions underneath the heading "Appreciate your Body".	"Looking after your body restores your Gift energy. What does your body need most right now?<br>• Sleep<br>• Exercise<br>• Food<br>• To be outside in nature	✓	✕	✓	✓	✕	✕
5	Nonplayer dialogue	2 nonplayer characters converse in the elevator.	Nonplayer 1: "Look! I got the Speed Gift! I didn't expect that. But I kinda love it."<br>Nonplayer 2: "Really? I'd be so embarrassed if I got that one…"<br>Nonplayer 1: "Well, that's just rude. We're all different!"	✕	✕	✕	✕	✓	✕
6	Interaction with trainers	Players are presented with opportunities to interact with the 6 trainers.	"You respect your body. That way, your Gift will function at its best."	✕	✓	✓	✓	✓	✕
7	Meditation mats	Players can interact with meditation mats, which contain 5 messages in the form of mantras.	"I will follow Flutter accounts that make me feel good".	✓	✓	✕	✕	✕	✕

8	Reaching the end of the game	3 possible endings contain dialogue from key characters.	"You've got a deal. Inez, Daniel come on! We have to get started right away! No more filters, no more bullying! Online or offline!"	✓	✓	✕	✓	✕	✓

[a]The first pop-up Flutter message was mandatory, requiring players to vote in agreement with a thumbs up or disagreement with a thumbs down. Players could only advance after voting.

[b]The remaining pop-up Flutter messages were nonmandatory. As such, players could continue playing regardless of whether they voted (ie, in agreement with a thumbs up or disagreement with a thumbs down) as was required with the first Flutter message.
