# Supplementary material for: Evaluating the Effectiveness of a Roblox Video Game (Super U Story) in Improving Body Image Among Children and Adolescents in the United States: Randomized Controlled Trial
Source: J Med Internet Res. 2025 Jul 31;27:e66625. doi: 10.2196/66625 (PMC12355144; doi:10.2196/66625)
Supplement: Multimedia Appendix 3 [file jmir_v27i1e66625_app3.pdf]

Multimedia Appendix – Time 2 Questionnaire

Welcome back! We're so happy you're here! 😊

Today, we'll ask you to play an online game. Before you continue, please make sure you are in a **quiet space, free from distractions.**

We'll also ask you to answer a few very short questions about how you're feeling today. As with our previous surveys, there are no right or wrong answers.

We will ask you questions **BEFORE** and **AFTER** you play the game.

It is important that you answer these questions immediately before you play the game and immediately after you've finished playing it. You will need about **40 minutes** to answer the questions and play the game.

If that sounds good and you're ready to get started, please click the arrow button to continue. ➡️

Which of these Roblox games have you played before?

- Break-in Story
- Tree Story
- Vacation Story
- Brookhaven
- Super U Story LOGIC: TERMINATE
- Daycare Story
- New Titanic Story
- Adopt Me
- None of the above

How much do you like playing these Roblox games?

	👎 I don't like it	👍/👎 It's OK	👍 I like it a little	👍👍 I like it a lot!	👍👍👍 I really really like it!!
LOGIC: INSERT GAMES HAVE PLAYED					

Our first task for you is to answer a few quick questions about how you feel RIGHT NOW, IN THIS MOMENT.

Before we begin, let's try an example question.

The example question is **"How happy do you feel about going to school, <u>RIGHT NOW</u>?"**

Your answer depends on how you are feeling. For instance,

- If you are not feeling happy about going to school, you might move the slider to ‘1’.
- If you are feeling a little happy about going to school, you might move the slider to ‘2’ or ‘3’.
- If you are feeling somewhat happy about going to school, you might move the slider to the number ‘5’.
- If you are feeling happy in general about going to school, you might move the slider to ‘7’.
- If you are feeling very happy about going to school, you might move the slider to ‘10’.

Okay, now you give it a try. Use the slider below and share **how happy you feel about going to school, <u>RIGHT NOW</u>**.

RESPONSES:

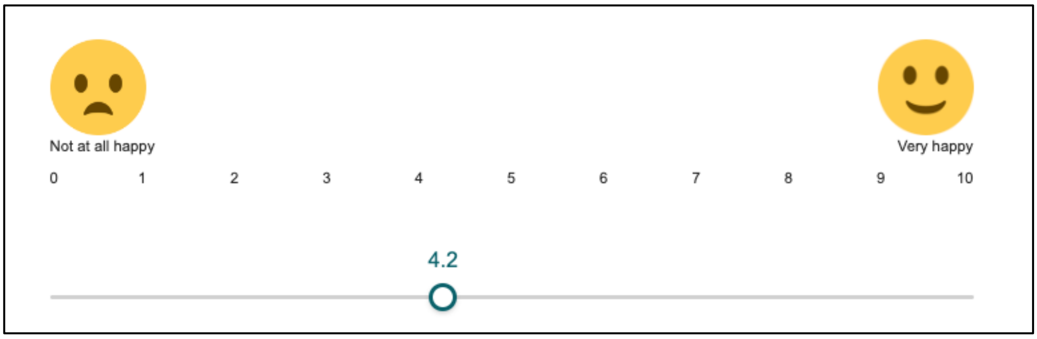

❓ If you still aren’t sure how to answer these kinds of questions, please ask your parent or guardian for help. You can also click on the “Survey Help” button at the bottom right-hand corner of the screen.

✓ If you understand how to answer this kind of question, please click the arrow button to continue.

Let's begin! 🚀 (And remember, the researcher will be available via the “Survey Help” button at the bottom right-hand corner of the screen in case you have any questions).

How happy do you feel about the way you look, **RIGHT NOW**?

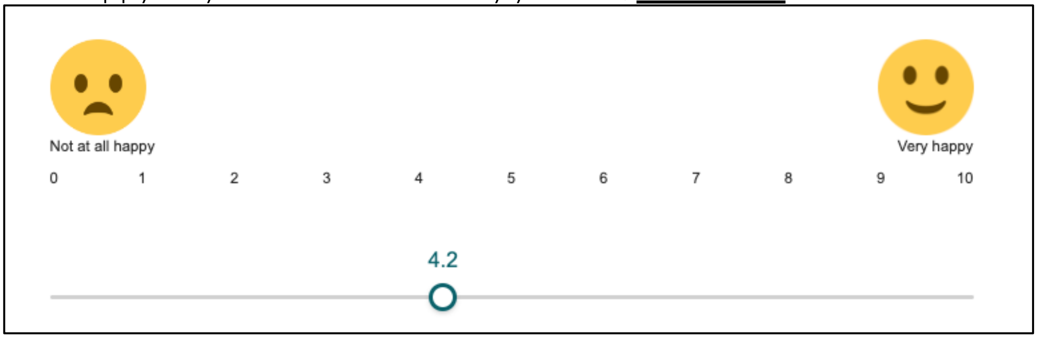

How happy do you feel about your body weight, **RIGHT NOW**?

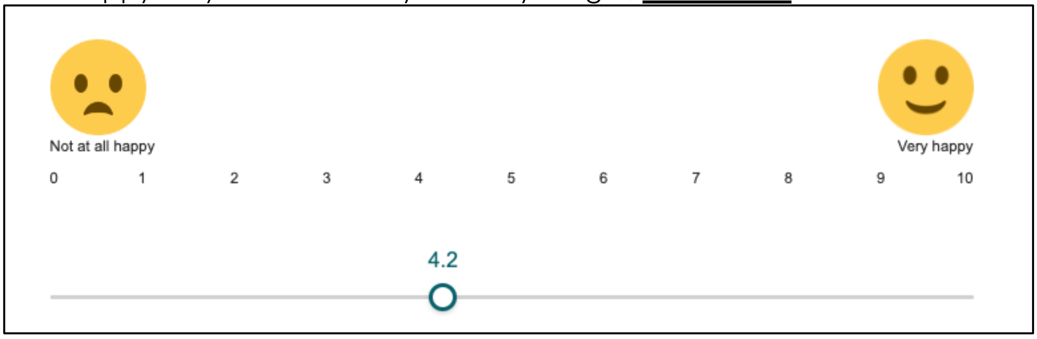

How happy do you feel about your body shape, **RIGHT NOW**?

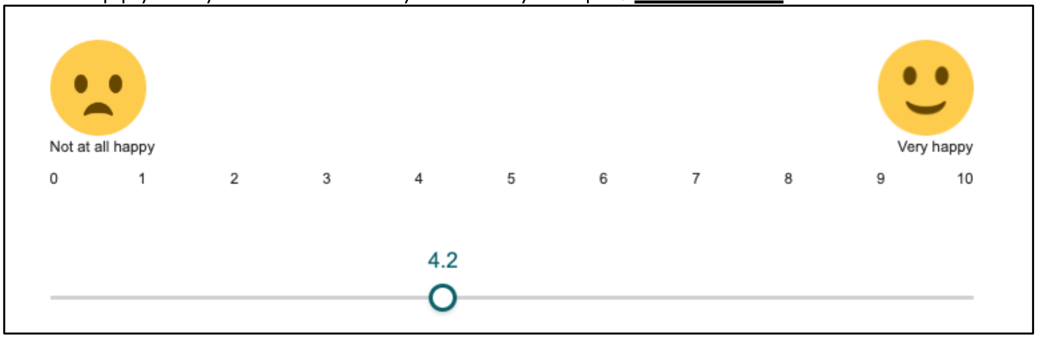

This scale ranges from ‘very sad’ to ‘very happy’. How do you feel, **RIGHT NOW**?

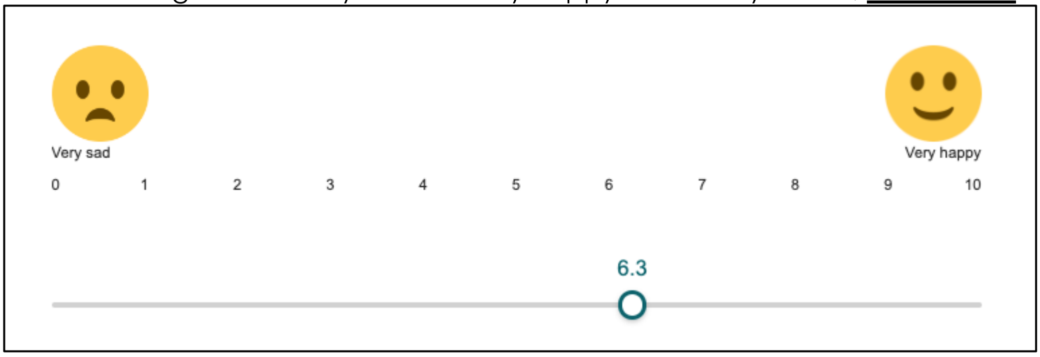

How happy do you feel with what your body can do, **RIGHT NOW**?

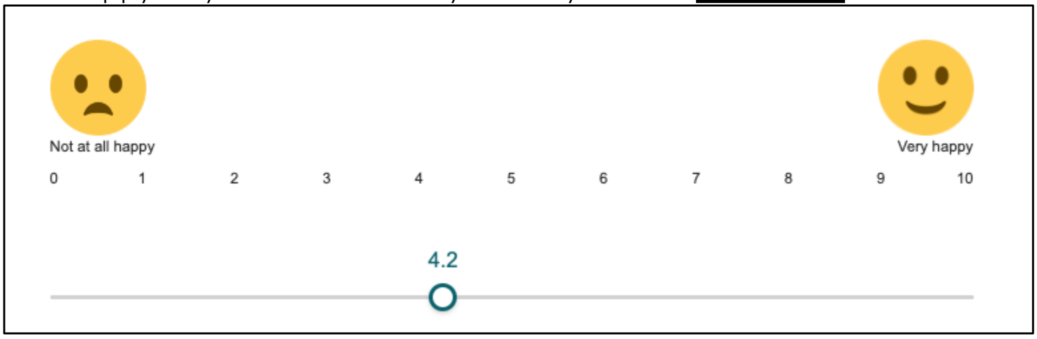

LOGIC: FOR INTERVENTION PARTICIPANTS

Now, it's time to play a game called **Super U Story**. It doesn't matter how well you do in the game. We just want you to play and have fun! 😁 Please play the game for at least 5 minutes and up to 30 minutes. (Please do not play longer than 30 minutes).

❗ **IMPORTANT: The link to Super U Story will open in another window. Click the white-and-green Play arrow and it will bring you to the Roblox platform for you to play it. Please DON'T close this survey when you go to play Super U Story. You will need to come back to this survey as soon as you're done playing the game**

📌 After you finish playing Super U Story in the app, **please remember to come back to this survey right after you're done** as we have a few more short questions for you to answer.

When you click the link to go to the game you will have to log in to your own Roblox account if you aren't already logged in on the device you're on.

Please enable pop-ups if they are disabled on your device.

🎮 Okay, time to get playing! 🎮

🔗 Here is the link for the game: https://www.roblox.com/games/9286287742/BETA-Super-U-Story

Enjoy!!

LOGIC: FOR ACTIVE CONTROL PARTICIPANTS

Now, it's time to play a game called **Rainbow Friends/Color Story**. It doesn't matter how well you do in the game. We just want you to play and have fun! 😁 Please play the game for at least 5 minutes and up to 30 minutes. (Please do not play longer than 30 minutes).

❗ **IMPORTANT: The link to Rainbow Friends Story (Color Story) will open in another window. Click the white-and-green Play arrow and it will bring you to the Roblox platform for you to play it. Please DON'T close this survey when you go to play Rainbow Friends Story (Color Story). You will need to come back to this survey as soon as you're done playing the game.**

📌 After you finish playing Rainbow Friends Story (Color Story) in the app, **please remember to come back to this survey in this web browser right after you're done** as we have a few more short questions for you to answer.

🎮 Okay, time to get playing! 🎮 When you click the link to go to the game you will have to log in to your own Roblox account if you aren't already logged in on the device you're on.

🔗 Here is the link for the game: https://www.roblox.com/games/9108988501/Rainbow-Friends-Story-Color-Story

Enjoy!!

LOGIC: FOR ATTENTION CONTROL PARTICIPANTS

Now, it's time to play **Animal Word Searches**. It doesn't matter how well you do. We just want you to play and have fun! 😁 Please play for at least 5 minutes and up to 30 minutes. (Please do not play longer than 30 minutes).

There are many different animal word searches to choose from. Please start with the category of **animals** that you're most interested in.

If you would like to make the word search **easier to complete**, please follow these 3 instructions:

1. Please click the settings button:

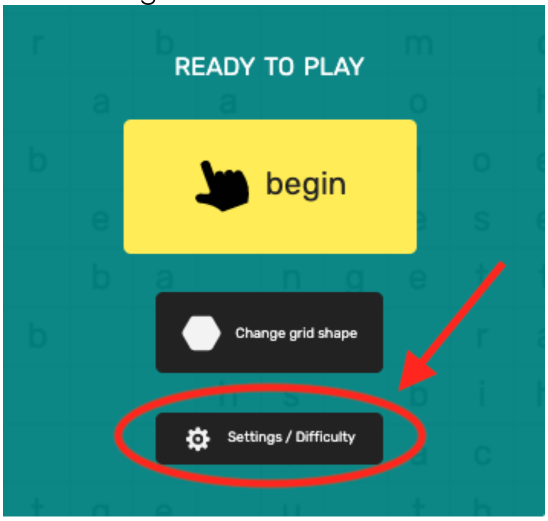

2. Then, click "KIDS MODE", and make sure it says "on":

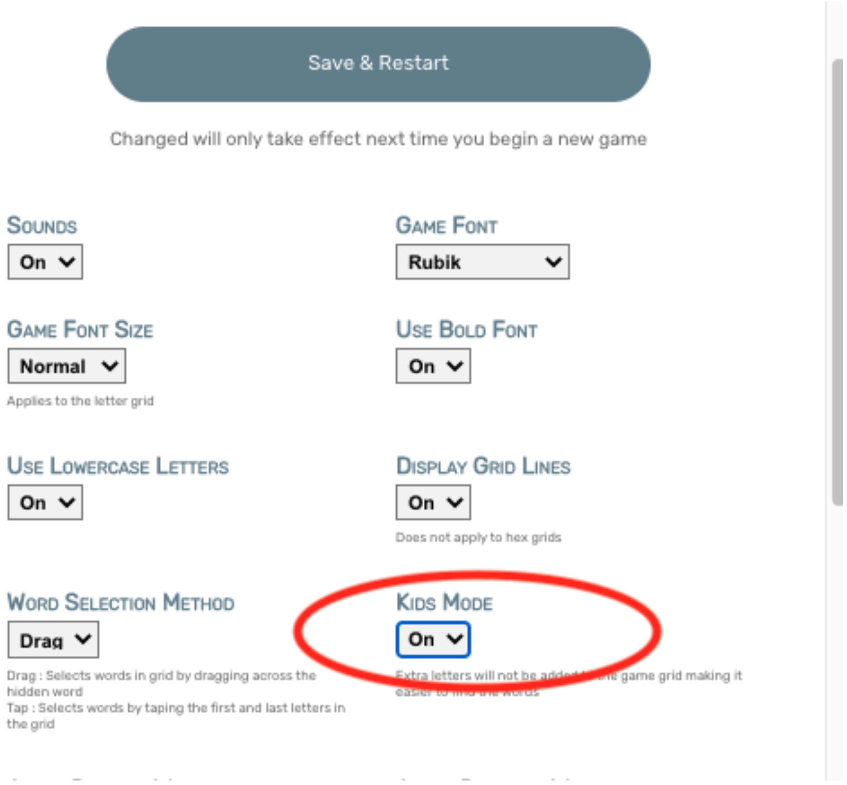

3. Then, click "save and restart":

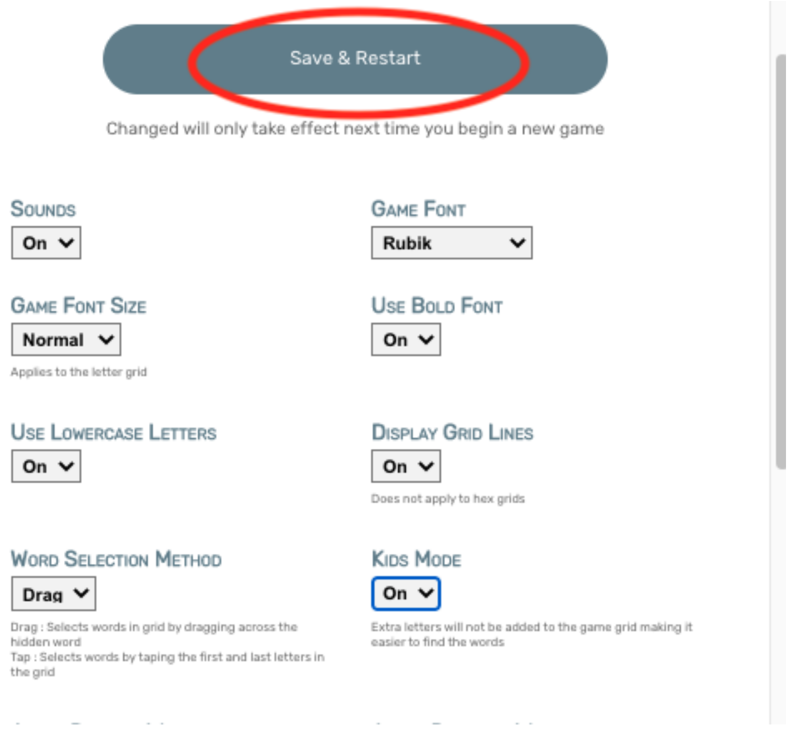

If you want harder animal word searches, make sure KIDS MODE says "off".

We would like you to do one or more animal word searches for up to 30 minutes. (Please do not play longer than 30 minutes).

If you finish your chosen word search and want to play more, **please use the BACK button to go back and choose another animal word search**:

You will NOT be judged on how many you complete. We just want you to have fun completing as many animal word searches as you can! 😁

❗ **IMPORTANT: The game will open in another window or tab. Please DON'T close this survey when you go to play the animal word searches.** ❗ **You will need to come back to this survey as soon as you're done playing the game(s).**

📌 After you finish doing animal word searches for 30 minutes, **please remember to come back to this survey** as we have a few more short questions for you to answer.

Okay, time to start searching!

🔗 Here is the link for the game: https://thewordsearch.com/cat/animals/

Enjoy!!

🙌 Thanks for playing! 🙌

You are almost finished with today's part of the research.

We have a few more very short questions for you to answer about how you are feeling **RIGHT NOW**, followed by LOGIC: a few questions about Super U Story / Rainbow Friends Story / Word searches.

Please click the arrow button to continue. ➡️

How happy do you feel about the way you look, **RIGHT NOW**?

RESPONSES:

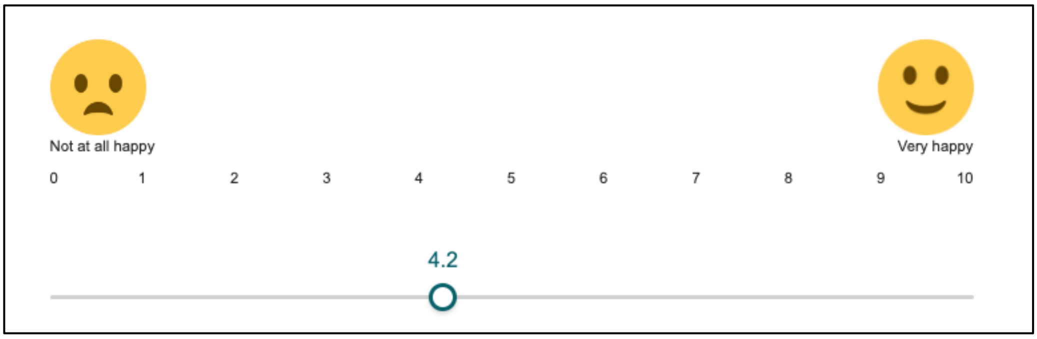

How happy do you feel about your body weight, **RIGHT NOW**?

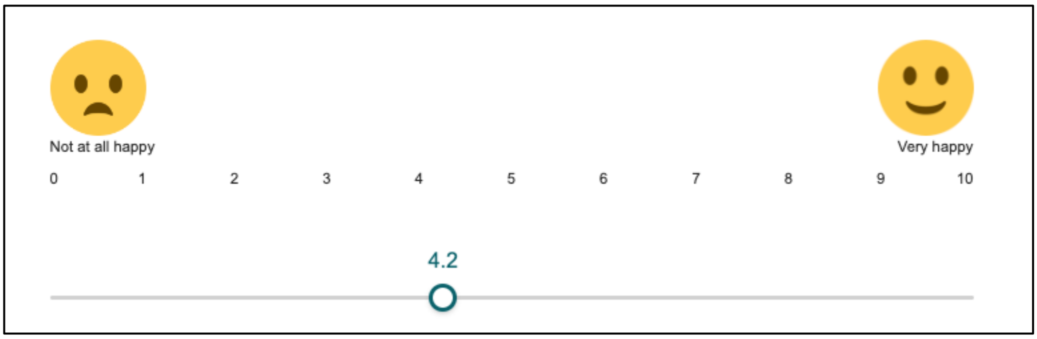

How happy do you feel about your body shape, **RIGHT NOW**?

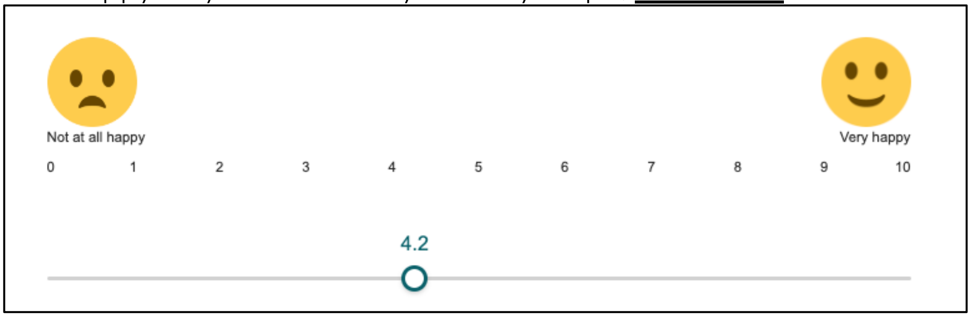

This scale ranges from 'very sad' to 'very happy'.

How do you feel, **RIGHT NOW**?

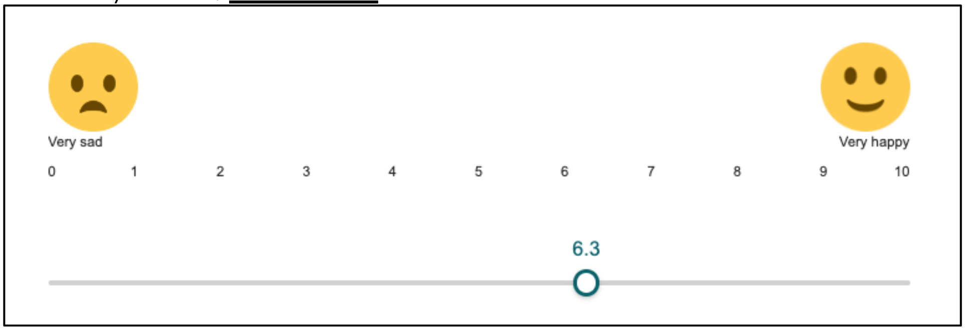

How happy do you feel with what your body can do, **RIGHT NOW**?

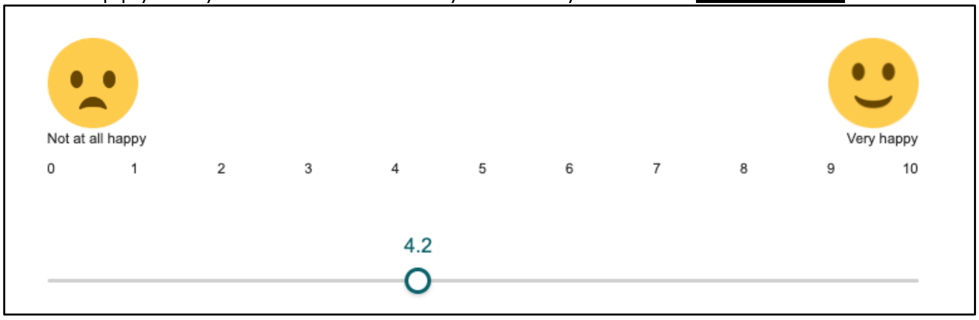

LOGIC: FOR INTERVENTION PARTICIPANTS ONLY

Now, we have some questions for you to help us understand what parts of Super U Story you interacted with.

Don't worry if you didn't interact with every part of the game. Your responses will help us understand what parts of the game are interesting to players.

Please click the arrow button to continue. ➡️

How long did you play Super U Story for?

- Less than 5 minutes
- 5-10 minutes
- 10-15 minutes
- 15-20 minutes
- More than 20 minutes
- I can't remember

What gift did you choose?

- Fire
- Flight
- Nature
- Strength
- Speed
- Water
- Don't remember

How many Flutter messages do you remember reading?

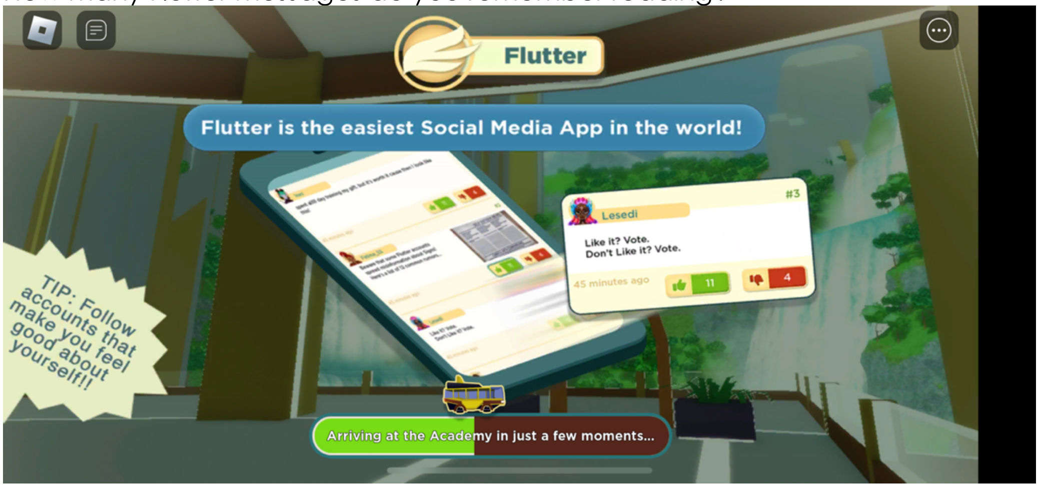

- 0 Flutter messages
- 1 Flutter message
- 2-3 Flutter messages
- 4-6 Flutter messages
- 7-10 Flutter messages
- 11-15 Flutter messages
- 16-20 Flutter messages
- More than 21 Flutter messages
- Not sure

Before traveling to the Academy, did you read the posters?

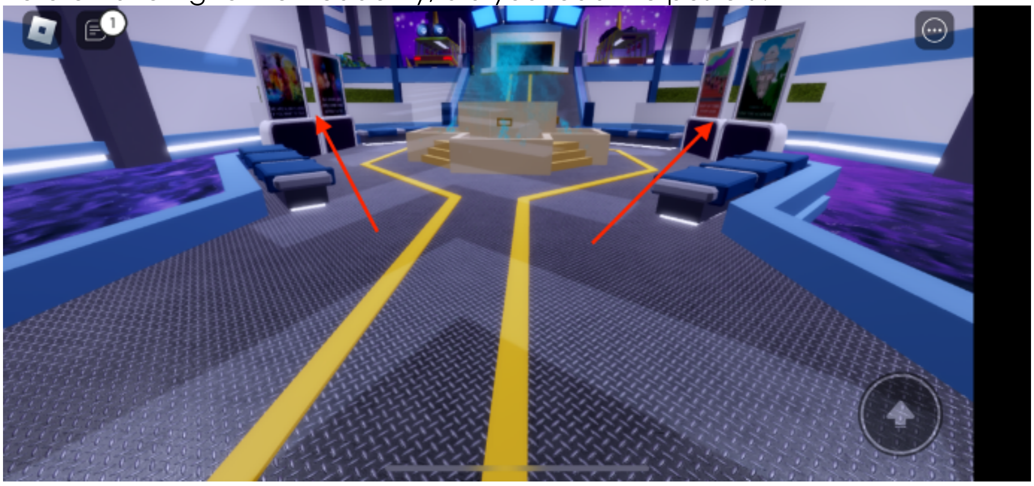

- Yes
- No
- Not sure

Before traveling to the Academy, did you interact with the Selfie Guy?

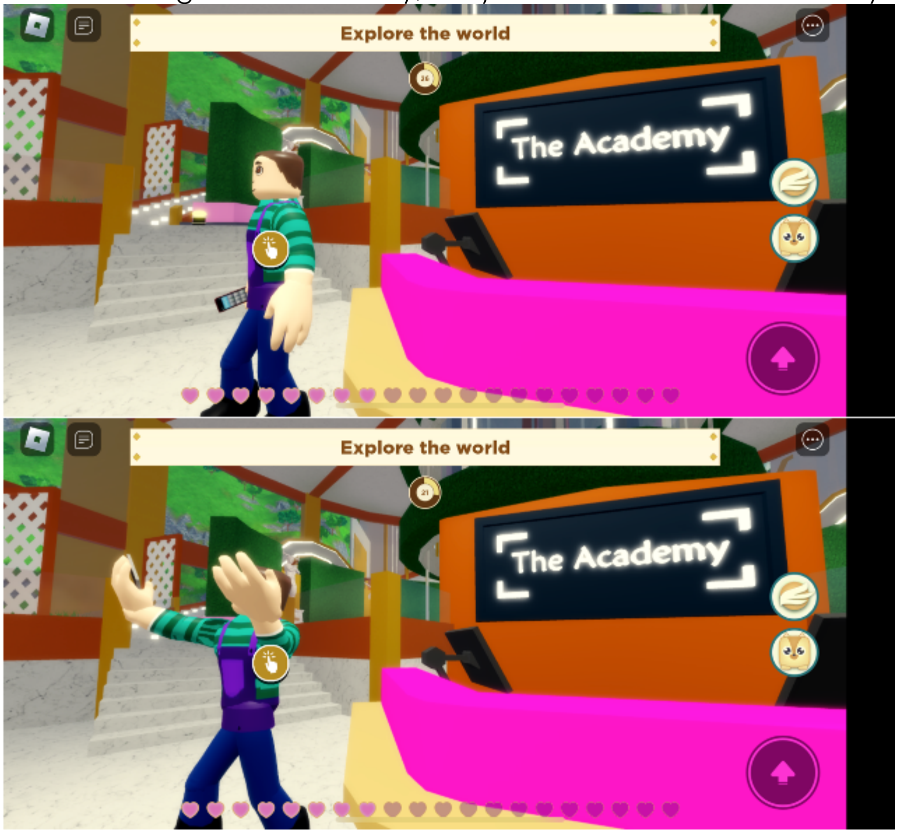

- Yes
- No
- Not sure

Before traveling to the Academy, did you read the news on the digital screens?

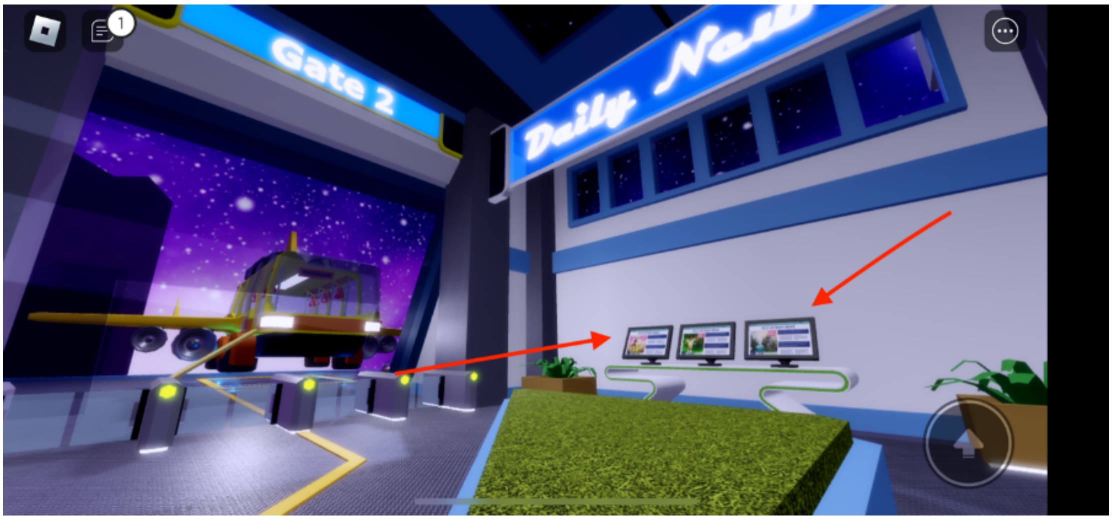

- Yes
- No
- Not sure

After arriving at the Academy, before getting on the elevator for the first time, did you interact with the screens in the Academy lobby?

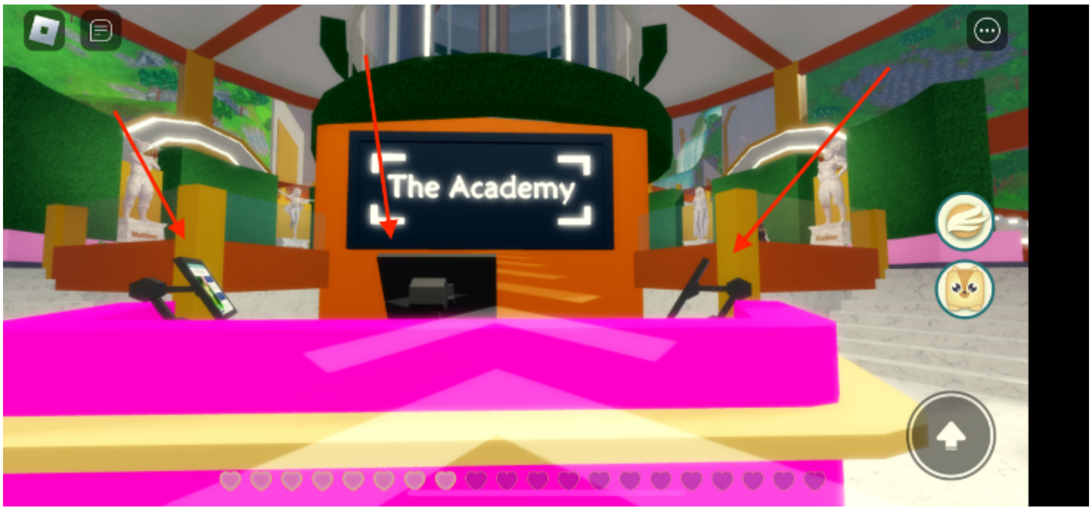

- Yes
- No
- Not sure

After arriving at the Academy, before getting on the elevator for the first time, did you interact with the trainer statues?

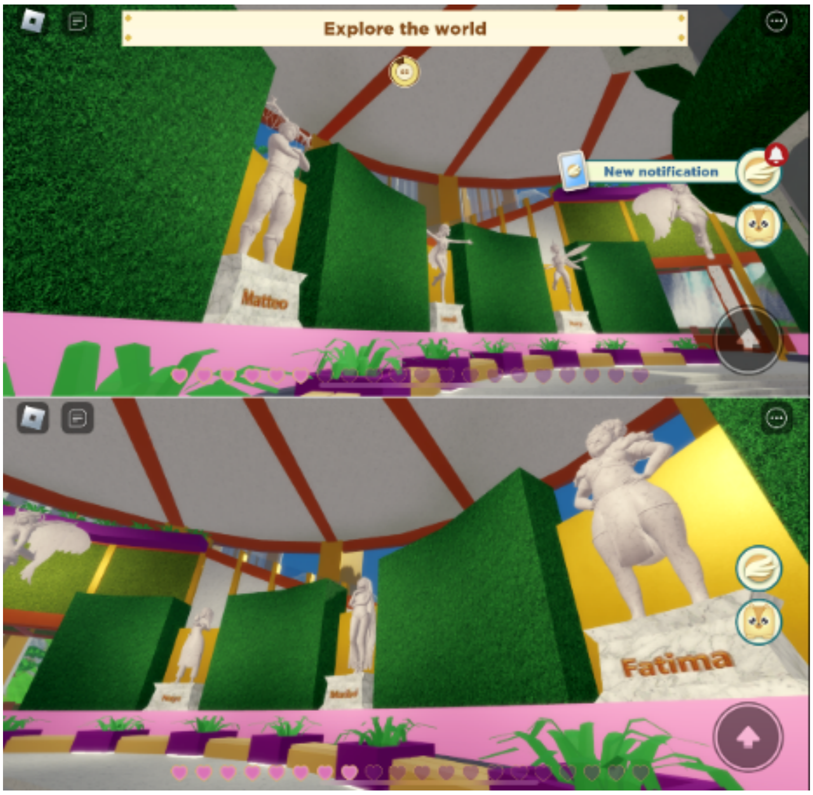

- Yes
- No
- Not sure

When you were in the elevator, did you read any of the conversations that were happening with the non-player characters (NPCs)?

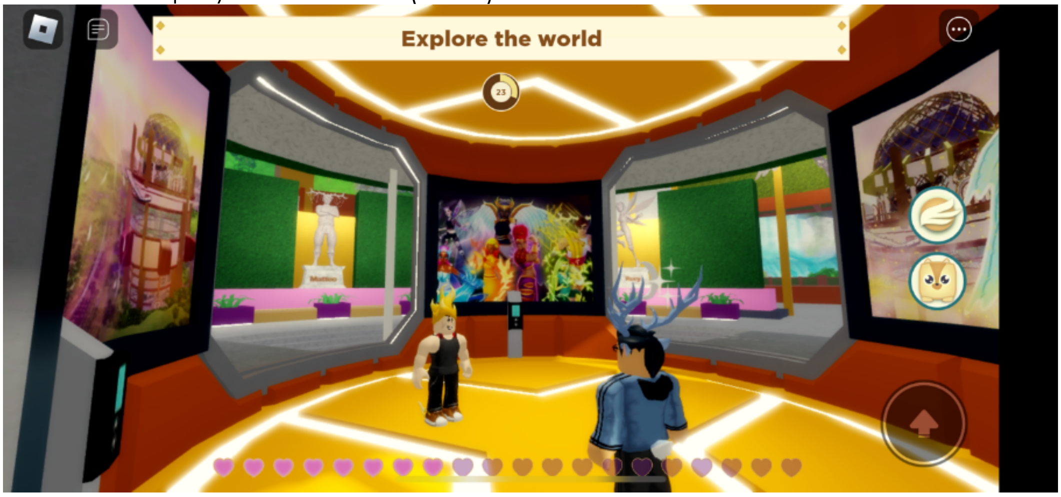

- Yes LOGIC ASK NEXT QUESTION
- No LOGIC: SKIP TO FOLLOWING QUESTION
- Not sure LOGIC: SKIP TO FOLLOWING QUESTION

How many conversations with the non-player characters (NPCs) in the elevator did you read?

- 1
- 2
- 3
- 4
- 5
- I can't remember

Did you interact with the other trainers?

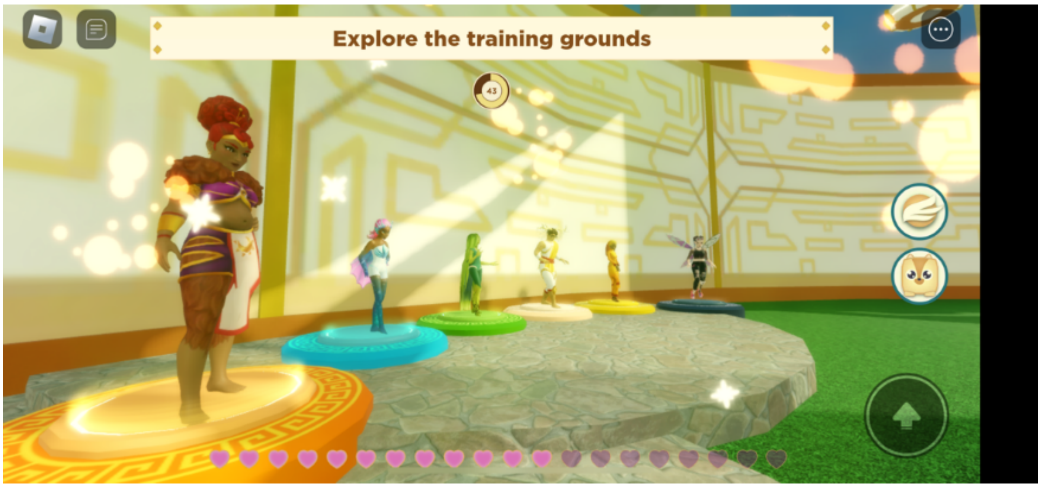

- Yes
- No
- Not sure

Did you interact with the food in the Dining Hall?

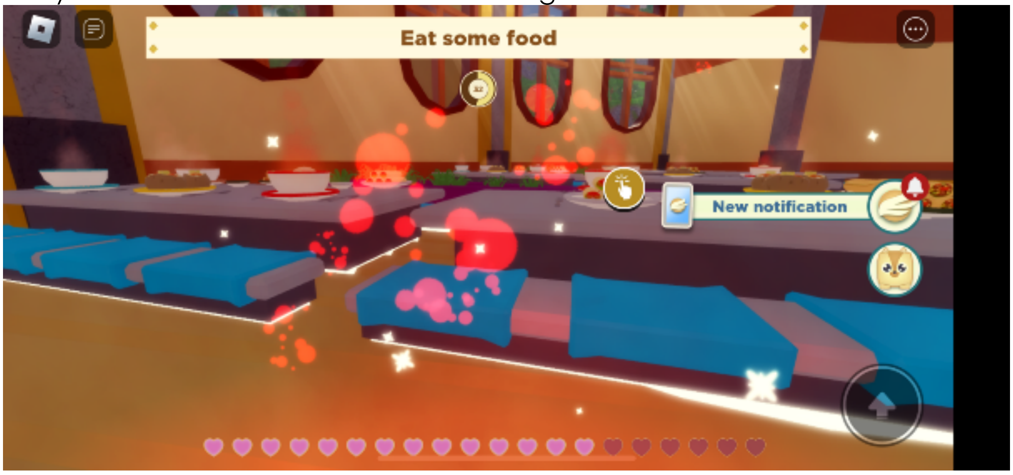

- Yes
- No
- Not sure

Did you interact with the meditation mats?

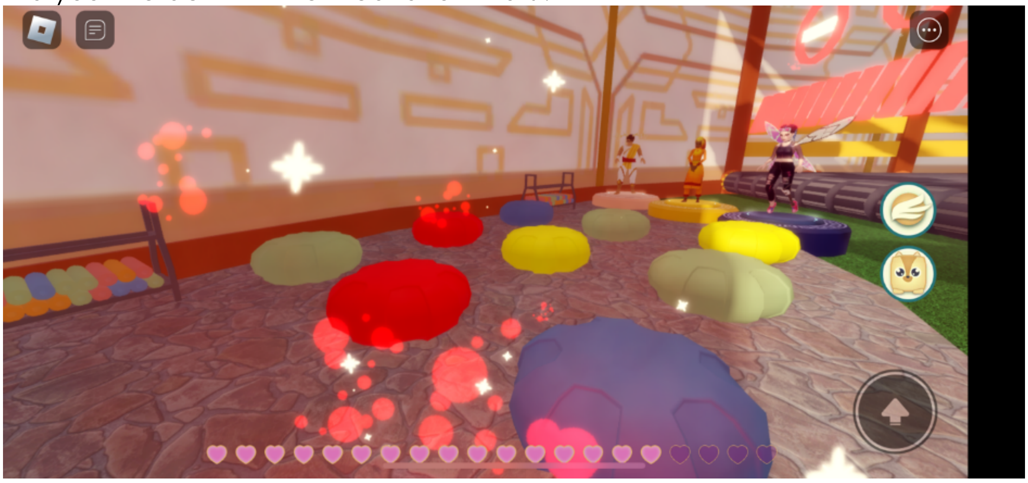

- Yes
- No
- Not sure

Did you get to the end of the game?

- Yes LOGIC: ASK NEXT QUESTION
- No LOGIC: SKIP TO ACCEPTABILITY QUESTIONS INTRO

Did you defeat Marzanna?

- Yes
- No LOGIC: SKIP TO ACCEPTABILITY QUESTIONS INTRO

Did you choose for Marzanna to leave the Academy?

- Yes LOGIC: SKIP TO ACCEPTABILITY QUESTIONS INTRO
- No
- Not sure

Did you choose for Marzanna to stay in the Academy?

- Yes
- No
- Not sure

Now we will ask you a few questions to help us understand what you liked and disliked about Super U Story.

Please click the arrow button to continue. ➡️

Please select how much you agree or disagree with each of the following statements.
We'd like you to click on the word that **best describes how much you agree or disagree with that sentence**.

	👎👎 Totally disagree	👎 Mostly disagree	👍/👎 Neither agree nor disagree	👍 Mostly agree	👍👍 Totally agree
I enjoyed playing Super U Story.					
I liked the story in Super U Story.					
I liked the Super U Story characters.					
I would recommend Super U Story to my friends.					

We would love to know a bit more about what you thought about Super U Story!

What did you **learn** from playing Super U Story? Please use the space below to share your answer:

What, if anything, did you **like** about Super U Story? Please use the space below to share your answer:

What, if anything, **didn't you like** about Super U Story? Please use the space below to share your answer:

🎉 Here are the FINAL questions!!

How did you travel to the training school?

- Swim
- Train
- Bus
- Plane
- Don't remember

Who is Marzanna?

- The headmistress of the school
- A trainer
- An angry and dangerous ex-student
- A helpful ex-student
- I don't know

LOGIC: FOR ACTIVE CONTROL PARTICIPANTS

How long did you play Rainbow Friends Story (Color Story)?

- Less than 5 minutes
- 5-10 minutes
- 10-15 minutes
- 15-20 minutes
- More than 20 minutes
- I can't remember

LOGIC: FOR ACTIVE CONTROL PARTICIPANTS

How did you travel to the bowling alley?

- Swim
- Train
- Bus
- Plane

LOGIC: FOR ACTIVE CONTROL PARTICIPANTS

After the bowling alley, you went to someone's house. Whose house was it?

- Maria's house
- Ethan's house
- Ravi's house
- Trevor's house

LOGIC: FOR ATTENTION CONTROL PARTICIPANTS

How much time did you spend doing animal word searches?

- Less than 5 minutes
- 5-10 minutes
- 10-15 minutes
- 15-20 minutes
- More than 20 minutes
- I can't remember

LOGIC: FOR ATTENTION CONTROL PARTICIPANTS

How many animal word searches did you complete?

- 1
- 2
- 3
- 4
- 5
- More than 5

LOGIC: FOR ATTENTION CONTROL PARTICIPANTS

Were the game instructions we provided clear?

- Yes LOGIC: SKIP TO TECHNICAL DIFFICULTY QUESTION
- No LOGIC: ASK NEXT QUESTION

Please explain why the instructions were not clear to you:

Did you experience any technical difficulties with the game (for example, being kicked out of the game, glitches, the screen freezing)?

- Yes LOGIC: ASK NEXT QUESTION
- No LOGIC: SKIP TO DIFFICULTY RETURNING TO SURVEY QUESTION

Please explain the technical difficulties you experienced:

Did you experience any difficulties returning to the survey after playing the game?

- Yes LOGIC: ASK NEXT QUESTION
- No LOGIC: SKIP TO FINAL MESSAGE

Please explain the difficulties you experienced retuning to the survey after playing the game:

🙏🏽 Thanks SO much for taking the time to play Super U Story/Rainbow Friends Story (Color Story) / animal word searches and answer all our questions! We REALLY appreciate it!

🗓️ We will be in touch in ONE week to ask you to complete our final survey.

👀 Keep an eye out for a message from the researcher!

❗ If answering these questions has upset you, please speak to your parent or caregiver, school counsellor, or family doctor ❗

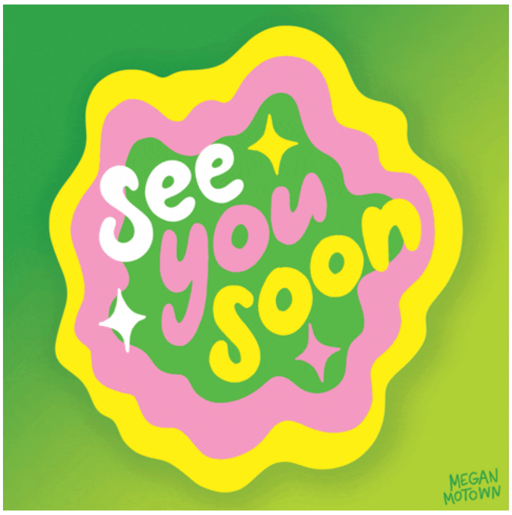

Please click on the arrow button on the bottom right to finish submitting your survey.
