# Supplementary material for: Evaluating the Effectiveness of a Roblox Video Game (Super U Story) in Improving Body Image Among Children and Adolescents in the United States: Randomized Controlled Trial
Source: J Med Internet Res. 2025 Jul 31;27:e66625. doi: 10.2196/66625 (PMC12355144; doi:10.2196/66625)

Multimedia Appendix – Time 3 Questionnaire

Welcome back! 👋

Just like the first survey you completed, this one asks questions about some of your everyday experiences and feelings about yourself and your body.

Before you start, here is a reminder of some important things we want you to know.

1. This is **not a test.** Everyone will have different answers, and there are no right or wrong answers.

2. It is really important that you **complete your survey privately.** If you have any questions, you may ask your parent or guardian for help or feel free to reach out to the researcher who will be happy to help you. You can click 'survey help' in the bottom right corner to contact the researcher.

3. All your answers are completely confidential – your parents, family, and friends won't see them.

4. If you don't feel comfortable answering a particular question, **just skip it** and go on to the next question. Some questions at the very beginning may ask you for a response and cannot be skipped.

5. **If you don't want to do the survey**, that's OK, you can stop at any point.

6. If you understand everything we've just told you, and you are happy to help us, **please click the arrow button to begin**.

Which of these Roblox games have you played before?

- Super U Story **LOGIC: TERMINATE**
- Break-in Story
- Tree Story
- Vacation Story
- Brookhaven
- Super U Story
- Daycare Story
- New Titanic Story
- Adopt Me
- None of the above

How much do you like playing these Roblox games?

	👎 I don't like it	👍/👎 It's OK	👍 I like it a little	👍👍 I like it a lot!	👍👍👍 I really really like it!!
**LOGIC: INSERT GAMES HAVE PLAYED**					

🔎 Before we get started with the next set of questions, let's refresh your memory on how to answer them.

⭐ You will need to select the answer that matches how you think and feel about important areas of your life. Remember, there are no right or wrong answers. ⭐

⭐ Here we have a sentence ("I like eating chocolate"). Beside the sentence, there are 5 single words that will stay the same:

"Never", "Rarely", "Sometimes", "Often", and "Always".

Please click on the word that **best describes how often you feel this way**.

For example, if you like eating chocolate **once in a while**, you would click on "rarely".

If you like eating chocolate **most of the time**, you would click on "often".

	Never	Rarely	Sometimes	Often	Always
I like eating chocolate					

Let's try another example.

Here we have a sentence ("I am a kind person"). Beside the sentence, there are 5 statements that will stay the same:

"Totally disagree", "Mostly disagree, "Neither agree nor disagree",
"Mostly agree", and "Totally agree".

How much do you agree or disagree with the following statement, "I am a kind person"?

For example, if you think you are **always** a kind person, click on "totally agree".

If you **don't** think you are a kind person **most of the time**, click on "mostly disagree".

❓ If you are unsure how to answer these kinds of questions, please ask your parent or guardian for help.

You can also click on the "Survey Help" button at the bottom right-hand corner of the screen.

✓ If you understand how to answer these kinds of questions, please click the arrow button to continue.

	👎👎 Totally disagree	👎 Mostly disagree	👍/👎	👍 Mostly agree	👍👍 Totally agree

			Neither agree nor disagree		
I am a kind person					

For this set of questions, we have a list with sentences on them. Just like the practice questions, there are 5 single words beside the sentences that will stay the same and read:

"Never", "Rarely", "Sometimes", "Often", and "Always".

We'd like you to click on each word that **best describes how often that sentence is true of you**.

This page has 21 different sentences, so please read each one carefully before you choose your answer. You are able to change your answer if you would like to.

	Never	Rarely	Sometimes	Often	Always
1. I like what I look like in pictures.					
2. Kids my own age like my looks.					
3. I'm pretty happy about the way I look.					
4. Most people have a nicer body than I do.					
5. My weight makes me unhappy.					
6. I like what I see when I look in the mirror.					
7. I wish I were thinner.					
8. There are lots of things I'd change about my looks if I could.					
9. Please click on "never".					
10. I'm proud of my body.					
11. I really like what I weigh.					
12. I wish I looked better.					
13. I often feel ashamed of how I look.					

14. Other people make fun of how I look.					
15. I think I have a good body.					
16. I look as nice as I'd like to.					
17. I often wish I looked like someone else.					
18. My looks upset me.					
19. I'm as nice looking as most people.					
20. My parents like my looks.					
21. I worry about the way I look.					

This set of questions asks what you think of your body. The options are

"Never", "Rarely", "Sometimes", "Often", And "Always".

Please choose the word which shows **how often you feel this way.**

Just like the last question you will click the word that best shows how often you feel this way.
This page has 10 sentences.

	Never	Rarely	Sometimes	Often	Always
1. I feel good about my body.					
2. I respect my body.					
3. I feel that my body has at least some good qualities.					
4. I take a positive attitude toward my body.					
5. I pay attention to what my body needs.					
6. I feel love for my body.					
7. I appreciate the different and unique things about my body.					
8. You can tell I feel good about my body by the way I behave.					
9. I am comfortable in my body.					
10. I feel like I am attractive even if I am different from pictures and videos of attractive people (e.g., models/actresses/actors).					

👏 Fantastic work! You've already completed more than half the survey! You have about 5 minutes left. Almost there! 🏁

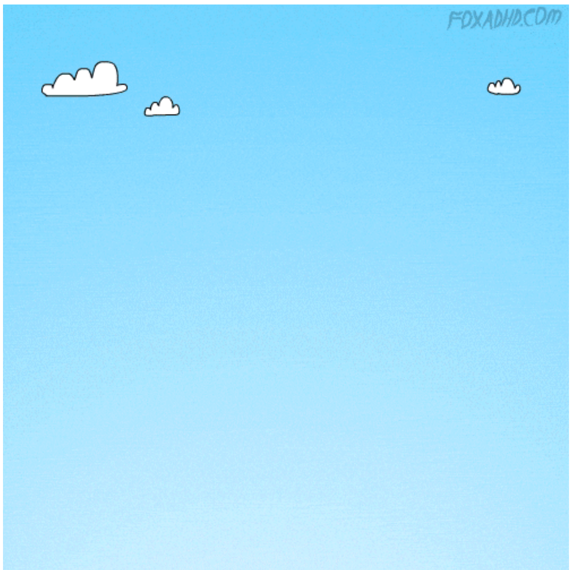

How much do you agree with the following statements?

The options are:

"Totally Disagree", "Mostly disagree", "Neither agree nor disagree", "Mostly agree", and "Totally agree".

Just like the earlier questions, please click the word that best describes how much you agree or disagree with the statement.

There are 13 sentences on this page.

	👎👎 Totally disagree	👎 Mostly disagree	👍/👎 Neither agree nor disagree	👍 Mostly agree	👍👍 Totally agree
1. I would like my body to look like the bodies of people who are on TV.					
2. I compare my body to the bodies of people who are on TV.					
3. I would like my body to look like the characters who appear in video games.					

4. I compare my appearance to the appearance of TV and movie stars.					
5. I would like my body to look like the people who are in movies.					
6. I compare my body to the bodies of people who appear in video games.					
7. Please click on “totally agree”.					
8. I wish I looked like the models in music videos.					
9. I compare my appearance to the appearances of people in video games.					
10. I try to look like the people on TV.					
11. I compare my body to the bodies of people who are on social media.					
12. I would like my body to look like the bodies of people who are on social media.					
13. I try to look like the people I see on social media.					

Do you look at social media on apps like Snapchat, Instagram, TikTok, Be Real, etc.?

- Yes
- No

Before we begin, let's try an example question.

The example question is **"I like going to the playground**"

Your answer depends on how much you agree or disagree with this statement. For instance,

- If you completely disagree and do not like going to the playground, you might move the slider to ‘1’.
- If you slightly disagree that you like going to the playground, you might move the slider to ’2’ or ‘3’.
- If you neither agree nor disagree that you like going to the playground, you might move the slider to the number ‘5’.
- If you slightly agree that you like going to the playground, you might move the slider to ‘7’.
- If you completely agree that you like going to the playground, you might move the slider to ‘10’.

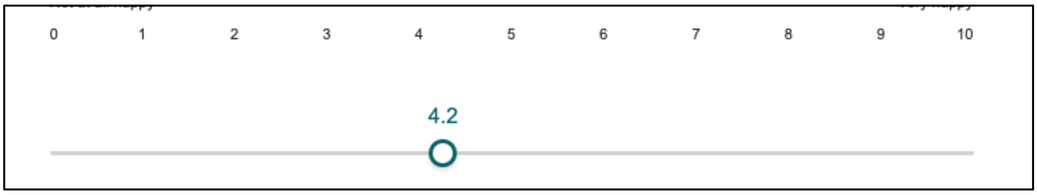

When I post on social media, it’s important to focus on what I’m doing, not what I look like.

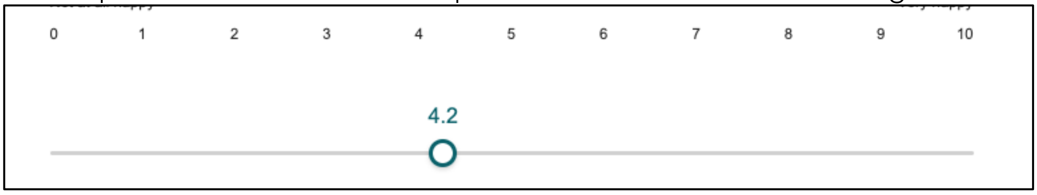

I would know what to do if I was being teased or bullied about my appearance on social media.

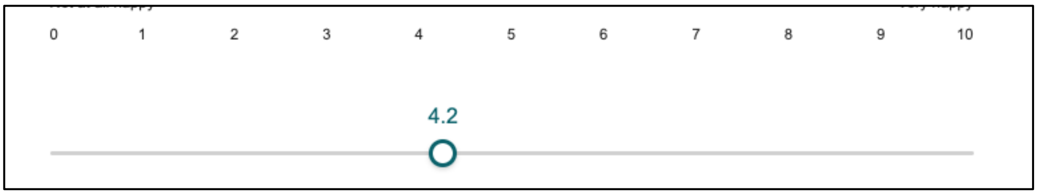

It is important to think before I accept everything I see on social media is true.

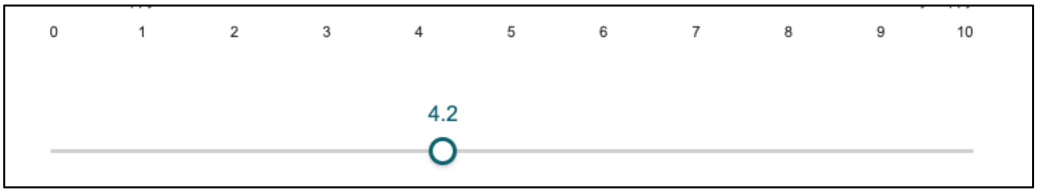

⭐ Thank you SO MUCH for your participation and helping us with this important work! ⭐

❗ *If answering these questions has upset you, please speak to your parent or carer, school counsellor or family doctor.* ❗

Please click the arrow button to close the survey. ➡️

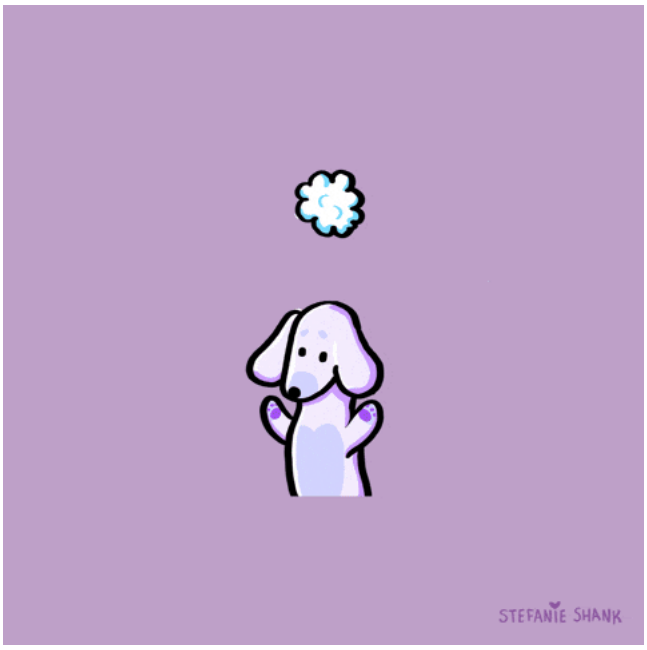

Supplement: Multimedia Appendix 4 [file jmir_v27i1e66625_app4.pdf]
