# Supplementary material for: Evaluating the Effectiveness of a Roblox Video Game (Super U Story) in Improving Body Image Among Children and Adolescents in the United States: Randomized Controlled Trial
Source: J Med Internet Res. 2025 Jul 31;27:e66625. doi: 10.2196/66625 (PMC12355144; doi:10.2196/66625)
Supplement: Multimedia Appendix 5 [file jmir_v27i1e66625_app5.pdf]

Multimedia Appendix – Content Analysis of Qualitative Acceptability Data

What did you learn?

Category	N (Responses)	%	Girls	%	Boys	%	Example Quote
**Total**	361	100	163	100	198	100	
			**Learnings related to key messaging**				
**The game taught me body appreciation/functionality**	23.00	6.37	9.00	5.52	14.00	7.07	To be thankful of what my body looks like (girl, age 11)
**The game taught me that you are unique or everybody's different**	20.00	5.54	13.00	7.98	7.00	3.54	The game shows how everyone has different gifts, just like humans are unique. I learned that I'm confident and brave. (girl, age 13)
**Learned to be yourself/believe in yourself**	26.00	7.20	13.00	7.98	13.00	6.63	To believe in ourselves, we are strong. (boy, age 12)
**Learned that body size and appearance don’t matter**	7.00	1.94	2.00	1.23	5.00	2.53	that it doesn't matter what you look like, just be yourself (boy, age 11)
**Social media literacy**	10.00	2.77	5.00	3.07	5.00	2.53	I learned how the game tries to urge to address unrealistic beauty ideals majority in social networks (girl, age 11)
**Learned to not bully/be mean to others**	9.00	2.49	5.00	3.07	4.00	2.02	I learned that making fun of others bodies is not cool (boy, age 12)
**Learned to not compare yourself to others**	5.00	1.39	3.00	1.84	2.00	1.01	Don't compare the way you look to the way other people look. (boy, age 11)

**Learned about being confident/self-esteem/feeling good about yourself**	5.00	1.39	4.00	2.45	1.00	0.51	I learned that Super U story is about teaching children how to be more confident and to love there self (girl, age 10)

Learnings NOT related to key messaging

**Category**	**N**	%	Girls	%	Boys	%	**Example Quote**
**The game taught me to defeat bad people**	15.00	4.16	5.00	3.07	10.00	5.05	I like this game very much and will fight off the bad guys with my friends to protect my school. (boy, age 11)
**The game taught me creativity and independence**	9.00	2.49	6.00	3.68	3.00	1.52	It allows me to gain more knowledge and improve my imagination. (girl, age 10)
**The game taught me how we should interact with others/be a good person**	11.00	3.05	2.00	1.23	9.00	4.55	I learned to communicate and cooperate. (girl, age 12)
**The game taught me persistence/hard work/perseverance**	11.00	3.05	0.00	0.00	11.00	5.56	I learned to persevere and never be afraid to push forward when things get tough (boy, age 11)
**Learned to be kind**	2.00	0.55	0.00	0.00	2.00	1.01	Be kind (boy, age 13)
**Learned about right and wrong**	5.00	1.39	1.00	0.61	4.00	2.02	Know right from wrong (boy, age 10)
**Liked the characters**	7.00	1.94	2.00	1.23	5.00	2.53	My favorite part was when you learned about the bully’s back story. (girl, age 10)
**Learned to not let others affect you**	6.00	1.66	3.00	1.84	3.00	1.52	That it does not matter what people say about you! (girl, age 11)

General comments about learnings							
**Category**	**N**	**%**	**Girls**	**%**	**Boys**	**%**	**Example Quote**
**Learned things about the game**	48.00	13.30	17.00	10.43	31.00	15.66	I learn how to move forward in the game. I learned i can talk to trainers and go to different places like the dining place. I can follow people and we can go places. (boy, age 11)
**The game changed my way of thinking**	1.00	0.28	0.00	0.00	1.00	0.51	Change our way of thinking (boy, age 10)
**The game taught me a lot**	11.00	3.05	8.00	4.91	3.00	1.52	Learned all kinds of new knowledge in this game. (boy, age 12)
**General comments**							
**The game taught me nothing**	40.00	11.08	23.00	14.11	17.00	8.59	I don't think i learned anything from the game. (girl, age 9)
**The game was overall positive/fun, happy/interesting**	59.00	16.34	26.00	15.95	33.00	16.67	I enjoyed the graphics and the gameplay of the characters I love the storyline as well you guys did a really great job when you came out with this game thank you I love roblux (boy, age 13)
**Didn't like the game**	9.00	2.49	7.00	4.29	2.00	1.01	I don't like games like this and I didn't know that before (girl, age 11)
**Didn't like the characters**	1.00	0.28	1.00	0.61	0.00	0.00	I didnt like the mean girls. And what they were saying (girl, age 13)
**Miscellaneous**	21.00	5.82	8.00	4.91	13.00	6.57	I don't know. (boy, age 12)

***N = Number of responses coded (not participants) as some participants provided data for multiple codes.**

What did you like?

Category	N	%	Girls	%	Boys	%	Example Quote
**Total**	365	100%	162	100%	203	100%	
				**Likes related to key messaging**			
**Liked the positive messages (Around body confidence, self-acceptance, self-love etc)**	37.00	10.14	23.00	14.20	14.00	6.90	As I said before, I liked the message I know a lot of teens my age are on every social media platform imaginable and that they dont like themselves because they see these super models with the perfect figure and doubt they can look like that, but you don't need to look like that, you just need to be yourself. It's a corny message, but a true one. (boy, age 13)
**Non-mandatory interactive elements**	7.00	1.92	3.00	1.85	4.00	1.97	i liked the characters especially selfie guy (girl, age 13)
				**Likes NOT related to key messaging**			
**Liked the characters**	66.00	18.08	32.00	19.75	34.00	16.75	I like the characters in it. (boy, age 10)
**Liked the powers/superhero aspects**	44.00	12.05	19.00	11.73	25.00	12.32	I really liked the powers and be able to pick them for myself. (girl, age 11)
**Liked the missions/tasks/obstacles**	19.00	5.21	5.00	3.09	14.00	6.89	I like the missions in the game. (boy, age 13)
**Liked interactive aspects or specific aspects of the game (bus or Academy or both)**	30.00	8.22	13.00	8.02	17.00	8.37	I like the bus ride and the Academy (boy, age 13)
**Liked the graphics**	17.00	4.66	6.00	3.70	11.00	5.42	I like the bright colors that make me want to play all the time. (Boy, age 12)

GENERAL likes/comments about the game							
**Category**	**N**	**%**	**Girls**	**%**	**Boys**	**%**	**Example Quote**
**Liked everything**	25.00	6.85	10.00	6.17	15.00	7.39	I like the colors of the game, i like the look of the game, with all the different objects, its supper cool. I also like that it gets more exciting after getting off the academy bus. So much more to look forward to. (boy, age 12)
**Thought the game was fun & Interesting**	31.00	8.49	14.00	8.64	17.00	8.37	It was fun and kept me occupied and engaged (boy, age 13)
**Liked the game because it was different**	3.00	0.82	2.00	1.23	1.00	0.49	Yes it was different story from the others. (girl, age 10)
**Thought the game was easy/understandable/well-guided**	11.00	3.01	5.00	3.09	6.00	2.96	I like how it tells you where to go and how to play so you don't get confused by anything, and you know what you are doing. (girl, age 13)
**Liked the overall story/specific parts of the story**	24.00	6.58	7.00	4.32	17.00	8.37	The story line makes me want to keep going (boy, age 9)
**Liked the positivity in the game**	8.00	2.19	4.00	2.47	4.00	1.97	i liked that it was possitive. (girl, age 12)
**Liked that the game encouraged you to be happy with yourself/express yourself**	6.00	1.64	0.00	0.00	6.00	2.96	I like how I can just be myself when I am playing the game. (boy, age 12)
**Miscellaneous responses**	15.00	4.11	7.00	4.32	8.00	3.94	The background music. (girl, age 11)
**Negative comments**							
**Category**	**N**	**%**	**Girls**	**%**	**Boys**	**%**	**Example Quote**
**Found the game difficult**	3.00	0.82	1.00	0.62	2.00	0.99	This game is a bit difficult. (boy, age 13)
**Did not like the game**	19.00	5.21	11.00	6.79	8.00	3.94	Not really but i think others would like it it just wasnt for me. (girl, age 11)

***N = Number of responses coded (not participants) as some participants provided data for multiple codes.**

What did you dislike?

Category	N	%	Girls	%	Boys	%	Example Quote
**Total**	356	100	158	100	198	100	
				**Disliked key messaging**			
**Disliked the messaging in the game/talking about body image**	9.00	2.53	4.00	2.53	5.00	2.54	I didn’t like that it was about body image and I didn’t like the use of pronouns. (boy, age 10)
**Felt the game was for girls**	2.00	0.56	0	0	2.00	1.02	It does feel a little like its for girls mostly. I do wish there was more stuff for boys. (boy, age 11)
				**Dislikes NOT related to key messaging**			
**Disliked characters/avatars**	31.00	8.71	11.00	6.96	20.00	10.2	I thought the characters were kinda ugly, this is mainly a Roblox thing since they decided introduce people to these generic boring human looking people and people are using the boring weird realistic humans instead of the charming iconic cute Roblox blocky guys. (boy, age 13)
**Had minor miscellaneous concerns about gameplay**	10.00	2.81	2.00	1.27	8.00	4.06	I don't like the food inside, it doesn't look delicious. (girl, age 11)
**Disliked the story and dialogue**	17.00	4.78	8.00	5.06	9.00	4.57	I didn't like that the dialogues were long and unskipable. (boy, age 11)
**Found the game boring**	15.00	4.21	7.00	4.43	8.00	4.06	It felt so slow and boring (girl, age 10)
**Had issues with obstacles in the game**	13.00	3.65	10.00	6.33	3.00	1.52	I didn't like the obby that was in the game MANY times. (girl, age 10)
				**General comments**			
**Disliked nothing or liked everything**	158.00	44.30	71.00	44.94	87.00	44.20	Nothing it was pretty cool. (boy, age 12)
**Dislikes related to mechanics of the game**	63.00	17.70	26.00	16.46	37.00	18.78	Hard to more around. lots of other players running fast. (boy, age 10)
**Miscellaneous responses**	24.00	6.74	9.00	5.70	15.00	7.61	It's too politically correct. (boy, age 10)
**Overall disliked the game**	12.00	3.37	9.00	5.70	3.00	1.52	it is just not the game for me. (girl, age 11)
**Found the game cringey**	2.00	0.56	1.00	0.63	1.00	0.51	Cringy. (boy, age 11)

***N = Number of responses coded (not participants) as some participants provided data for multiple codes.**
