# Supplementary material for: Evaluating the Effectiveness of a Roblox Video Game (Super U Story) in Improving Body Image Among Children and Adolescents in the United States: Randomized Controlled Trial
Source: J Med Internet Res. 2025 Jul 31;27:e66625. doi: 10.2196/66625 (PMC12355144; doi:10.2196/66625)
Supplement: Multimedia Appendix 6 [file jmir_v27i1e66625_app6.pdf]

Multimedia Appendix

The following table gives the *P* value for the potential moderating effect of age and gender for all outcomes.

**Outcome**	**Moderator**	
	**Age**	**Gender**
Body Satisfaction	.080	.389
Mood	.550	.629
Body Functionality	.584	.524
Body Esteem	.597	.557
Body Appreciation	.770	.337
SATAQ	.776	.968
SML1	.956	.937
SML2	.702	.197
SML3	.773	.385
