# Supplementary material for: Evaluating the Effectiveness of a Roblox Video Game (Super U Story) in Improving Body Image Among Children and Adolescents in the United States: Randomized Controlled Trial
Source: J Med Internet Res. 2025 Jul 31;27:e66625. doi: 10.2196/66625 (PMC12355144; doi:10.2196/66625)
Supplement: Multimedia Appendix 7 [file jmir_v27i1e66625_app7.pdf]

Multimedia Appendix – Intention-to-treat analyses

The following summarizes the intention-to-treat (ITT) analyses.

		Time 2a versus Time 2b			
Measure	Comparison	*F*	*df*1, *df*2	*P*	η^2
Body satisfaction	Omnibus	1.229	2, 1439	.293	.002
	I[a] versus AC[b]	1.929	1, 958	.165	.002
	I versus AttC[c]	1.885	1, 960	.170	.002
Mood	Omnibus	4.506	2, 1438	.011	.006
	I versus AC	0.001	1, 957	.972	.000
	I versus AttC	6.304	1, 960	.012	.007
Body functionality	Omnibus	1.179	2, 1439	.308	.002
	I versus AC	1.051	1, 958	.306	.001
	I versus AttC	2.026	1, 960	.155	.002
		Time 1 versus Time 3			
Body esteem	Omnibus	6.245	2, 1359	.002	.009
	I versus AC	8.561	1, 910	.004	.009
	I versus AttC	0.042	1, 913	.837	.000
Body appreciation	Omnibus	4.771	2, 1355	.009	.007
	I versus AC	5.750	1, 906	.017	.006
	I versus AttC	0.092	1, 912	.762	.000
Internalization	Omnibus	4.081	2, 1355	.017	.006
	I versus AC	2.220	1, 906	.137	.002
	I versus AttC	1.869	1, 912	.172	.002
Social media literacy: Item 1	Omnibus	0.646	2, 1354	.524	.001
	I versus AC	1.420	1, 905	.234	.002
	I versus AttC	0.318	1, 911	.573	.000
Social media literacy: Item 2	Omnibus	3.510	2, 1355	.030	.005
	I versus AC	6.476	1, 906	.011	.007
	I versus AttC	0.613	1, 912	.434	.001
Social media literacy: Item 3	Omnibus	0.840	2, 1353	.432	.001
	I versus AC	1.597	1, 904	.207	.002
	I versus AttC	0.718	1, 911	.317	.001

[a]Intervention group.
[b]Active control group.
[c]Attention control group.
